# Sirtuins in Medicine: Multifaceted Roles in Physiological Processes and Cardiovascular Diseases

**DOI:** 10.3390/biom16060793

**Published:** 2026-05-28

**Authors:** Jan Krekora, Oliwia Matuszewska-Brycht, Jerzy Krzysztof Wranicz, Michał Krejca, Krzysztof Kaczmarek, Piotr Merks, Jarosław Drożdż

**Affiliations:** 1 2nd Department of Cardiology, Medical University of Lodz, 92-213 Lodz, Poland; 2 Department of Electrocardiology, Medical University of Lodz, 92-213 Lodz, Poland; 3 Department of Pharmacology and Clinical Pharmacology, Faculty of Medicine, Collegium Medicum, Cardinal Stefan Wyszynski University in Warsaw, 01-938 Warsaw, Poland

**Keywords:** sirtuins, SIRT1, SIRT3, SIRT6, aging, cardiovascular disease, NAD^+^ metabolism, oxidative stress, mitochondrial dysfunction, inflammation, cardiac remodeling, atherosclerosis

## Abstract

Sirtuins are an evolutionarily conserved family of nicotinamide adenine dinucleotide (NAD^+^)-dependent enzymes that regulate aging, cellular stress responses, and metabolic homeostasis. In mammals, seven isoforms (SIRT1–SIRT7) differ in subcellular localization, substrate specificity, and enzymatic activity, allowing them to control genomic stability, mitochondrial function, redox balance, inflammation, apoptosis, autophagy, and cell proliferation. Increasing evidence links sirtuin dysregulation to age-related chronic diseases, particularly cardiovascular disease (CVD). This review provides an integrated overview of the structure, enzymatic functions, localization, and biological specialization of mammalian sirtuins, with an emphasis on their roles in physiological aging and cardiovascular homeostasis. We discuss the involvement of individual sirtuins in major cardiovascular pathologies, including metabolic cardiomyopathy, myocardial ischemia–reperfusion injury (IRI), cardiac hypertrophy, fibrosis, heart failure, atherosclerosis, coronary artery disease, and hypertension. Particular focus is placed on SIRT1, SIRT3, and SIRT6, which emerge as key regulators of endothelial integrity, mitochondrial quality control, oxidative stress, inflammatory signaling, and myocardial remodeling. We also highlight the context-dependent and sometimes dual effects of other sirtuin isoforms in CVD. Finally, we summarize current therapeutic strategies targeting sirtuins, including activators, NAD^+^-boosting approaches, and selective inhibitors, and discuss the main challenges for future clinical translation in cardiovascular medicine, including precision, isoform-specific intervention design strategies, and long-term clinical implementation.

## 1. Introduction

Aging and the development of chronic degenerative diseases are closely associated with a progressive loss of cellular homeostasis and reduced resilience to environmental and intrinsic stressors [1]. The sirtuin family of nicotinamide adenine dinucleotide (NAD^+^)-dependent enzymes has emerged as a key regulator of aging, metabolism, and age-related disorders. Interest in sirtuins began with the identification of the silent information regulator 2 gene, SIR2, in *Saccharomyces cerevisiae* in the 1970s [2]. Initially described as a mating-type regulator, SIR2 was later shown to mediate transcriptional silencing at ribosomal DNA, telomeres, and silent mating-type loci. In the late 1990s, genetic studies established Sir2 as a conserved longevity factor, with deletion shortening lifespan, and overexpression extending replicative lifespan in yeast and promoting longevity in *Caenorhabditis elegans* [3,4]. Subsequent research identified Sir2 as an NAD^+^-dependent histone deacetylase, linking cellular redox status to chromatin regulation, genomic silencing, and aging [5]. The discovery of Sir2 homologues across species led to the designation of this enzyme family as sirtuins. In mammals, seven homologues (SIRT1–SIRT7) have been identified. Among them, sirtuin 1 (SIRT1) is the most extensively studied and represents the closest mammalian homologue of yeast Sir2 [6].

Sirtuins regulate multiple cellular processes, including DNA repair, mitochondrial function, glucose and lipid metabolism, oxidative stress responses, apoptosis, and inflammation [1,7]. They are recognized as key modulators of aging through their roles in cellular senescence, genomic maintenance, nutrient sensing, protein homeostasis, and circadian regulation [1,8]. Moreover, sirtuins integrate metabolic and nutritional signals. For example, SIRT1 regulates hepatic gluconeogenesis and glycolysis during fasting, thereby coordinating metabolic adaptation with age-related processes [9]. Dysregulation of sirtuin expression or activity has been implicated in several chronic conditions, including cardiovascular disease and cancer, making them attractive therapeutic targets [10]. Recent studies have further clarified their role in cardiovascular biology. Both reduced expression and impaired activity of SIRT1 are associated with endothelial dysfunction and vascular aging, key contributors to cardiovascular (CV) risk [11]. Sirtuins modulate critical pathological pathways in the CV system, including oxidative stress, apoptosis, cellular senescence, metabolic imbalance, DNA damage, and mitochondrial dysfunction [12]. Among them, SIRT1 plays an important role in reverse cholesterol transport and atherogenesis, influences mitochondrial biogenesis, antioxidant defenses, and inflammatory signaling in cardiovascular cells [13]. Recent evidence suggests that SIRT1 exerts context-dependent effects on cardiac energy metabolism, with both protective and potentially deleterious consequences in cardiovascular disease (CVD) [14].

Given the central role of sirtuins in CV homeostasis and pathology, this review provides an integrated overview of the mammalian SIRT family in the context of CVD. It discusses their contribution to genomic integrity, metabolic and redox balance, stress resistance, and aging, and evaluates the therapeutic potential and current limitations of sirtuin-targeted interventions in CV medicine.

## 2. Structure and Enzymatic Activity of Sirtuins

Sirtuins are a conserved family of NAD^+^-dependent enzymes found across species, from bacteria to mammals, where they regulate genome stability, energy metabolism, and cell cycle regulation [1]. Their defining feature is a conserved catalytic core of approximately 250–275 amino acids, comprising an inverted Rossmann-fold domain that binds NAD^+^ and a zinc-binding domain [15,16]. In contrast, the N- and C-terminal regions show substantial variability, which determines subcellular localization, protein–protein interactions, and isoform-specific regulatory properties [15].

Human sirtuins are grouped into four classes: class I (SIRT1–3), class II (SIRT4), class III (SIRT5), and class IV (SIRT6–7) based on phylogenetic relationships. Despite this diversity, all share a conserved catalytic mechanism requiring NAD^+^ as a cosubstrate. During lysine deacylation, NAD^+^ is cleaved by sirtuins to generate nicotinamide and an acyl-ADP-ribose species. This mechanism distinguishes them from class I and II histone deacetylases [17]. Catalysis involves sequential substrate and NAD^+^ binding, glycosidic bond cleavage, and acyl-group transfer, resulting in deacylated lysine and by-products [1]. Although initially characterized as deacetylases, mammalian sirtuins display a broad spectrum of enzymatic activities. SIRT1, SIRT2, SIRT3, and SIRT7 function primarily as NAD^+^-dependent deacetylases, whereas SIRT4 acts mainly as an ADP-ribosyltransferase regulating glutamate dehydrogenase, thereby modulating insulin secretion [18,19]. SIRT5 preferentially catalyzes desuccinylation, demalonylation, and deglutarylation, while SIRT6 exhibits deacetylase and mono-ADP-ribosyltransferase activities, together with potent long-chain defatty-acylase activity [20,21]. Consistent with this functional diversity, sirtuins remove a broad spectrum of acyl modifications from histone and non-histone proteins and can catalyze ADP-ribosylation, thereby modulating protein activity, stability, subcellular localization, and interaction networks [5,22,23].

## 3. Localization and Functional Specialization of Sirtuins

The functional diversity of sirtuins reflects their distinct subcellular localization, which determines substrate accessibility and responsiveness to metabolic cues. Although all sirtuins share a conserved catalytic domain, their compartmentalization across the nucleus, cytoplasm, and mitochondria enables isoform-specific regulation of chromatin dynamics, metabolism, and stress responses. Each isoform functions within specialized biochemical niches [1].

SIRT1, SIRT6, and SIRT7 are predominantly nuclear. SIRT1 is mainly localized in the nucleus but can shuttle to the cytosol depending on cell type, developmental stage, or stress exposure [24]. Cytosolic localization of SIRT1 has been associated with altered apoptotic sensitivity in certain cell lines [25] and has been observed in subsets of neurons and ependymal cells [24]. In the nucleus, SIRT1 regulates transcription and metabolic adaptation through deacetylation of key factors, including FOXO3a, peroxisome proliferator-activated receptor alpha (PPARα), peroxisome proliferator-activated receptor gamma coactivator 1-alpha (PGC-1α), nuclear factor-κB (NF-κB), and sterol regulatory element-binding protein 1 (SREBP1), thereby coordinating oxidative stress responses, lipid and glucose metabolism, and inflammatory signaling [1,26]. SIRT1 also modulates insulin secretion, maintains organelle homeostasis, and exerts anti-inflammatory and antiapoptotic properties, partly through the regulation of p53 activity [27,28].

SIRT6 is enriched on chromatin where it regulates transcription, DNA repair, and metabolic homeostasis. It deacetylates histone marks such as histone H3 lysine 9 (H3K9) and H3 lysine 56 (H3K56), thereby controlling chromatin accessibility and genome stability [29]. SIRT6 also contributes to systemic glucose and lipid homeostasis [30]. Its deficiency leads to severe genomic instability, premature aging phenotypes, and metabolic deterioration, whereas its overexpression has been shown to extend lifespan in mice [31]. This broad physiological impact has positioned SIRT6 as a promising therapeutic target, particularly for age-related and neurodegenerative disorders [32].

SIRT7 is predominantly localized to the nucleolus, where it forms part of the RNA polymerase I transcriptional machinery and promotes ribosomal RNA transcription [33]. In hepatic experimental models, SIRT7 has been reported to repress Myc-dependent transcriptional programs and attenuate endoplasmic reticulum stress and fatty liver pathology; this should not be generalized as a universal role in cellular stress resistance [34]. It can also reduce hypoxia-inducible factor 1-alpha (HIF-1α) and HIF-2α protein levels through a mechanism independent of prolyl hydroxylation and canonical proteasomal or lysosomal degradation, suggesting a noncanonical role in hypoxic signaling [35]. In chromatin regulation, SIRT7 functions as a histone desuccinylase contributing to chromatin compaction and DNA double-strand break repair [36]. Altered SIRT7 activity has been implicated in cardiorenal hypertrophy, fibrosis, remodeling, heart failure, atherosclerosis, and renal dysfunction, although available evidence indicates context-dependent rather than uniformly protective or harmful effects [37].

SIRT2 is the only sirtuin whose steady-state localization is primarily cytoplasmic, although it can shuttle between the cytoplasm and nucleus [38]. In the cytoplasm, SIRT2 deacetylates substrates such as α-tubulin, FOXO1, and the gluconeogenic enzyme phosphoenolpyruvate carboxykinase, linking it to microtubule dynamics, adipocyte differentiation, gluconeogenesis, and metabolic adaptation [38]. During mitosis, SIRT2 associates with chromatin and deacetylates histone H4K16, supporting chromosome condensation. Evidence further connects SIRT2 with the regulation of the anaphase-promoting complex/cyclosome, DNA damage responses, genome integrity, and tumor-suppressive mechanisms [39]. SIRT2 also has tissue-specific functions, including the regulation of hepatic lipid metabolism and roles in oligodendrocyte and myelin biology [38,40].

SIRT3, SIRT4, and SIRT5 are primarily mitochondrial isoforms that regulate lysine acylation and mitochondrial metabolic enzyme activity, thereby influencing substrate use, adenosine triphosphate (ATP) production, and reactive oxygen species (ROS) handling [41,42]. SIRT3 is the principal mitochondrial deacetylase, activating key enzymes involved in the tricarboxylic acid cycle, fatty-acid β-oxidation, ketogenesis, and antioxidant defense, including superoxide dismutase 2 (SOD2) [43]. SIRT3 is abundantly expressed in metabolically active tissues such as brown adipose tissue, heart, skeletal muscle, and brain, where its deacetylase activity supports mitochondrial function under conditions of energetic stress [44]. In contrast, SIRT4 acts mainly as a metabolic brake: it restrains selected mitochondrial catabolic pathways by modulating glutamate dehydrogenase, glutamine metabolism, fatty-acid oxidation, insulin secretion, and redox balance, but its effects vary by tissue and disease context [45,46]. SIRT5 functions predominantly as a desuccinylase, demalonylase, and deglutarylase, modifying enzymes in the tricarboxylic acid cycle, β-oxidation, the urea cycle, oxidative phosphorylation, mitochondrial respiration, thermogenesis, and redox balance [47]. Although SIRT4 and SIRT5 are broadly expressed, they exert particularly prominent effects in tissues with high oxidative capacity, such as the liver, heart, skeletal muscle, and kidney, where they regulate fuel selection, urea formation, and ROS handling [48].

Under metabolic stress conditions, such as fasting or caloric restriction, rising NAD^+^ levels activate nuclear, cytosolic, and mitochondrial sirtuins, initiating coordinated transcriptional and posttranslational programs that promote mitochondrial homeostasis, antioxidant defense, and anti-inflammatory signaling [49]. These features position sirtuins, through their localization-specific enzymatic activities, as central nodes linking cell-intrinsic energy status to chromatin regulation, organelle function, and ultimately, aging trajectories. Table 1 summarizes subcellular localization, enzymatic activities and principal cellular functions of mammalian sirtuins.

## 4. Role of Sirtuins in Physiological Processes

### 4.1. Genomic Stability and DNA Damage Responses

Aging is accompanied by a gradual decline in genome maintenance, and several sirtuins, especially SIRT1 and SIRT6, orchestrate DNA repair, chromatin organization, and stress-induced transcriptional changes [50,51]. SIRT1 redistributes on chromatin after DNA damage and supports DNA repair while helping to restrain age-related transcriptional dysregulation [52]. SIRT6 exerts a complementary protective role in genome maintenance: it deacetylates histone H3K9 and other chromatin substrates at sites of DNA damage, promotes the recruitment or stabilization of repair factors, and protects against genomic instability and progeroid phenotypes [53]. SIRT6 also contributes to telomere maintenance in vascular smooth muscle cells (VSMCs).

Mitochondrial sirtuins may influence genome stability indirectly by controlling mitochondrial metabolism and redox state. In human mesenchymal stem cells, reduced SIRT3 expression increases susceptibility to oxidative stress, whereas SIRT3 overexpression enhances antioxidant defense, reduces oxidative-stress-associated senescence, and improves cellular survival [54]. These findings support a role for sirtuins in limiting DNA damage accumulation and cellular senescence, but the strength and direction of these effects vary by isoform, cell type, and experimental model.

### 4.2. Sirtuins in the Regulation of Inflammatory Pathways and Their Relevance to Aging

The contribution of sirtuins to physiological aging is closely linked to their regulation of inflammatory signaling networks. Chronic, low-grade inflammation, commonly referred to as “inflammaging”, is driven by persistent activation of innate immune pathways such as NF-κB, tumor necrosis factor alpha (TNF-α) signaling, and the NOD-like receptor family pyrin domain-containing 3 (NLRP3) inflammasome [55]. Members of the sirtuin family modulate these pathways through effects on transcription, protein acetylation, and mitochondrial stress responses; however, the direction and magnitude of these effects are sirtuin-, tissue-, and model-dependent [1].

NF-κB acts as a master regulator of inflammatory gene expression, controlling cytokines, chemokines, adhesion molecules, and inflammasome-related genes [56]. After stimulation by proinflammatory cytokines such as TNF-α, interleukin-1β (IL-1β), or IL-6, NF-κB translocates to the nucleus, where its transcriptional activity is further regulated by posttranslational modifications, including acetylation of the RelA/p65 subunit. SIRT1 negatively regulates this pathway by deacetylating RelA/p65 at lysine 310, thereby reducing NF-κB-dependent transcription in experimental models [56,57]. Reduced SIRT1 activity may therefore facilitate NF-κB-driven inflammatory gene expression, although this relationship should be interpreted in a cell- and disease-specific manner [1]. SIRT6 also represses NF-κB signaling, but through a chromatin-based mechanism: it associates with RelA and deacetylates histone H3K9 at NF-κB target-gene promoters, limiting inflammatory transcription and contributing to the aging-like phenotype observed in Sirt6-deficient mice [58].

SIRT7 appears to exhibit context-dependent behavior. In cultured cells, SIRT7 deacetylates Ran at Lys37 and promotes nuclear export of NF-κB p65, thereby limiting nuclear p65 accumulation [59]. A study of cisplatin-induced acute kidney injury reported that Sirt7 deficiency reduced TNF-α expression, NF-κB nuclear signaling, renal inflammation, and tubular injury, suggesting that SIRT7 can also support inflammatory responses in specific disease settings [60]. These findings indicate that SIRT7 should not be described as uniformly anti-inflammatory or pro-inflammatory.

SIRT1, SIRT3, and SIRT5 have been implicated in inflammasome regulation, although the evidence is pathway- and model-specific. In hepatic IRI, SIRT1 attenuated NLRP3 inflammasome activation through a miR-182/XBP1-dependent mechanism, reducing IL-1β and IL-18 production [61]. SIRT3 can attenuate mitochondrial ROS-dependent NLRP3 inflammasome assembly and activation under nutrient-stress conditions by activating SOD2; however, this effect should not be generalized to all inflammasomes, because SIRT3-mediated deacetylation of NLRC4 has also been reported to promote NLRC4 inflammasome activation [62]. Thus, available evidence supports an indirect, context-dependent protective role for SIRT3 against mitochondrial ROS-driven NLRP3 activation, rather than a direct or universal inhibitory effect on inflammasome signaling. Through suppression of NF-κB, SIRT1 and SIRT6 can reduce the expression of proinflammatory mediators, including TNF-α, although these effects remain cell- and stimulus-dependent [56,57,63]. In endothelial cells, reduced SIRT7 levels are associated with increased expression of TNF-α, IL-1β, and IL-6, whereas SIRT7 overexpression reduces cytokine release [1]. SIRT1 and SIRT6 similarly contribute to the attenuation of TNF-α-driven inflammation through both transcriptional and epigenetic mechanisms [57,64].

By regulating NF-κB activity, inflammasome signaling, mitochondrial ROS, and cytokine production, sirtuins act as key modulators of the inflammatory landscape that shapes aging. Their anti-inflammatory actions may help preserve mitochondrial function, proteostasis, and genomic stability, but these effects should be interpreted according to tissue, disease model, and individual sirtuin. Other mitochondrial sirtuins also modulate inflammation. In a mouse model of traumatic spinal cord injury, SIRT4 impaired the anti-neuroinflammatory activity of infiltrating regulatory T cells by downregulating AMPK and limiting Treg differentiation, thereby promoting neuroinflammatory responses [65]. In contrast, SIRT5 suppresses macrophage IL-1β production by desuccinylating and activating pyruvate kinase M2, and protects mice against dextran sulfate sodium-induced colitis [66].

Sirtuin-dependent regulation of inflammatory signaling may also be relevant to neuroinflammation. In microglial models of amyloid-β toxicity, SIRT1 inhibited NF-κB signaling and reduced microglia-dependent neuronal injury [67]. These findings support a role for selected sirtuins in modulating inflammatory responses during aging-related tissue stress, but they are derived largely from experimental models. Therefore, therapeutic targeting of sirtuin pathways in inflammaging should account for tissue type, inflammatory stimulus, disease stage, and the specific sirtuin involved.

### 4.3. Sirtuins as Key Regulators of Metabolic Homeostasis and Their Implications for Aging

Central metabolic pathways, including glucose, lipid, and amino acid metabolism, are regulated by nutrient- and stress-responsive regulatory proteins, among which sirtuins have emerged as key modulators. Because metabolic inflexibility is a hallmark of aging, experimental evidence that sirtuins regulate mitochondrial function, insulin signaling, and substrate utilization supports a mechanistic link between sirtuin activity and age-related metabolic decline [1,68]. This link is particularly relevant to insulin resistance, which is usually accompanied by compensatory hyperinsulinemia and has been described as an underestimated, often clinically silent contributor to cardiometabolic disease in both developed and developing populations. Since insulin resistance may precede overt hyperglycemia by years, affected individuals can carry increased cardiovascular risk before diabetes is diagnosed [69,70,71]. These abnormalities overlap with pathways regulated by SIRT1, SIRT3, and SIRT6, which modulate insulin signaling, mitochondrial oxidative metabolism, redox balance, and inflammatory responses in experimental models [72,73,74,75]. Thus, sirtuins may represent relevant modulators of early cardiometabolic dysfunction, although direct clinical evidence that targeting sirtuins prevents insulin resistance-associated cardiovascular disease remains limited.

SIRT1 is one of the best-characterized sirtuin regulators of systemic glucose homeostasis. AMP-activated protein kinase (AMPK) increases cellular NAD^+^ availability and thereby enhances SIRT1 activity, whereas SIRT1 supports AMPK-dependent metabolic adaptations, including mitochondrial oxidative metabolism [72]. In insulin-resistant cells and tissues, SIRT1 improves insulin sensitivity at least partly by repressing protein tyrosine phosphatase 1B (PTP1B), a negative regulator of insulin receptor signaling [73,76]. This supports a role for SIRT1 in insulin signaling, although its metabolic effects vary according to tissue type, nutritional state, and disease context.

Other sirtuins also regulate glucose metabolism. SIRT6 contributes to systemic glucose regulation through hepatic and peripheral mechanisms. In mice, physiological SIRT6 overexpression enhanced insulin sensitivity in the liver and skeletal muscle [74], whereas hepatic SIRT6 suppressed gluconeogenesis through activation of GCN5 and subsequent acetylation-dependent inhibition of PGC-1α [26]. SIRT1, SIRT3, and SIRT6 can therefore counter several metabolic defects associated with insulin resistance in experimental models, but current evidence does not establish altered sirtuin activity as a universal primary cause of human insulin resistance [72,73,74,75].

Sirtuins also influence lipid metabolism. In SIRT1-transgenic mice, SIRT1 deacetylated cAMP response element-binding protein (CREB) at Lys136, thereby reducing its phosphorylation and altering hepatic lipid accumulation and secretion [77]. In liver and metabolic disease models, SIRT1 has also been reported to promote fatty acid oxidation and reduce lipid deposition through activation of AMPK- and transcription factor-dependent mechanisms [1,28]. SIRT2 protects against hepatic steatosis in experimental models by deacetylating hepatocyte nuclear factor-4α [78]. Mitochondrial sirtuins play important roles in fatty acid oxidation. During fasting, SIRT3 enhances β-oxidation by deacetylating long-chain acyl-CoA dehydrogenase (LCAD) and other mitochondrial enzymes [79,80]. SUMOylation of SIRT3 reduces its catalytic activity, whereas SUMOylation-deficient SIRT3 increases mitochondrial deacetylation and fatty acid utilization, illustrating an additional layer of mitochondrial metabolic control [81]. By contrast, SIRT4 suppresses fatty acid oxidation by repressing malonyl-CoA decarboxylase, thereby favoring lipid storage over mitochondrial lipid catabolism in the models studied [82]. SIRT5 regulates mitochondrial lysine acylation and can modify fatty acid oxidation enzymes; separate adipocyte studies suggest that SIRT5 may also restrain adipocyte differentiation through AMPK-associated mechanisms, although these findings come from distinct experimental systems [47,83,84].

Amino acid metabolism becomes particularly important during nutrient stress, and mitochondrial sirtuins regulate glutamine utilization, anaplerosis, insulin secretion, and redox balance [1,47,80]. Loss of SIRT3 increases glutamine flux into nucleotide biosynthesis through mTORC1-dependent mechanisms in SIRT3-deficient cancer-cell models, suggesting a shift toward anabolic metabolism under mitochondrial stress [85]. SIRT4 suppresses glutamine catabolism by ADP-ribosylation and inhibition of glutamate dehydrogenase, thereby reducing amino acid-stimulated insulin secretion in pancreatic β-cells [86]. SIRT4 also limits glutamine entry into the tricarboxylic acid cycle after DNA damage and functions as a tumor suppressor in this metabolic context [87,88]. SIRT5 enhances antioxidant defense by desuccinylating isocitrate dehydrogenase 2 and deglutarylating glucose-6-phosphate dehydrogenase, thereby supporting NADPH production and reduced glutathione levels [89]. Overall, mitochondrial sirtuins respond to nutritional stressors such as fasting and caloric restriction, conditions that extend lifespan in several model organisms [80,90]. SIRT3 deficiency impairs mitochondrial energy metabolism, fatty acid oxidation, and metabolic stress tolerance [75,91], whereas SIRT4 and SIRT5 influence insulin secretion, glutamine use, lipid handling, and redox homeostasis in a tissue- and context-dependent manner [47,86,89].

### 4.4. Sirtuins, Reactive Oxygen Species, and Mitochondrial Antioxidant Defense

Sirtuins regulate cellular responses to oxidative stress by modifying mitochondrial enzymes, antioxidant transcriptional networks, and NAD^+^-dependent stress pathways. These functions are relevant to aging because age-associated tissues often show increased oxidative damage and reduced capacity to maintain redox homeostasis, although the magnitude and mechanisms vary among tissues and disease states [92,93,94]. Mitochondrial sirtuins are particularly important in this context. SIRT3 is the best-characterized mitochondrial antioxidant sirtuin. In calorie restriction and stress models, it deacetylates and activates manganese superoxide dismutase (MnSOD/SOD2), thereby enhancing mitochondrial superoxide detoxification and reducing ROS accumulation [95]. SIRT3 deficiency results in mitochondrial protein hyperacetylation and has been linked experimentally to impaired mitochondrial redox control, metabolic dysfunction, vascular oxidative stress, and hypertension-related endothelial injury [75,91,96,97]. SIRT5 complements SIRT3 by regulating nicotinamide adenine dinucleotide phosphate (NADPH)-dependent antioxidant capacity [89]. It desuccinylates isocitrate dehydrogenase 2 (IDH2) and deglutarylates glucose-6-phosphate dehydrogenase (G6PD), increasing NADPH production, maintaining reduced glutathione, and lowering susceptibility to oxidative stress in cellular models [89]. SIRT4 has a more context-dependent role [1,88]. In clear cell renal cell carcinoma models, its overexpression enhances ROS production and sensitizes cells to apoptosis while suppressing HIF-1α/heme oxygenase-1 (HO-1) signaling, indicating that SIRT4 can amplify oxidative stress under specific oncogenic conditions [98]. Sirtuins also interact with nuclear stress-response pathways that regulate antioxidant capacity, mitochondrial biogenesis, and metabolic adaptation [1,99]. SIRT1 is required for several resveratrol-induced metabolic effects in mice, including AMPK activation and improved mitochondrial function, although AMPK–SIRT1 signaling is stimulus- and tissue-dependent [100]. SIRT1 also regulates antioxidant gene expression in vascular endothelial cells through a FOXO3a/peroxisome proliferator-activated receptor-γ coactivator-1α (PGC-1α) complex, linking deacetylation-dependent transcriptional control to oxidative-stress resistance [101]. Because FOXO activity declines in several age-related pathologies, maintenance of SIRT1–FOXO3 signaling is likely an important component of stress-resilient aging. SIRT3 augments FOXO3a-dependent antioxidant defense in cardiac hypertrophy models, increasing expression of MnSOD and catalase and reducing ROS-dependent hypertrophic signaling [102]. SIRT6 has similarly been reported to protect cardiomyocytes during IRI by enhancing FoxO3α-dependent antioxidant defenses [103]. These findings support crosstalk among sirtuins, AMPK, FOXO proteins, and PGC-1α, but the direction and strength of these interactions depend on the experimental system. Nuclear factor erythroid 2-related factor 2 (Nrf2) orchestrates antioxidant response element-driven transcription of detoxifying and antioxidant enzymes, including HO-1 and other phase II defense genes [104,105]. Evidence for direct sirtuin–Nrf2 regulation is strongest in defined disease models rather than as a universal mechanism. For example, SIRT6 inhibited inflammatory and oxidative responses in vascular endothelial cells through NRF2-dependent signaling [63], while SIRT1/Nrf2/HO-1 signaling has been implicated in neuronal and vascular protection in experimental oxidative-stress models [104,105,106]. Therefore, sirtuin–Nrf2 pathways should be described as context-specific antioxidant mechanisms rather than as a generalized property of all sirtuins.

PGC-1α is a master regulator of mitochondrial biogenesis and oxidative metabolism [101]. It also induces SIRT3 expression, and SIRT3 can feed back on mitochondrial redox homeostasis by activating SOD2 [95]. SIRT6 also interacts with AMPK–PGC-1α signaling in metabolically active tissues such as the kidney and heart models, where it supports mitochondrial function and stress resistance [1,63,103,107]. Together, these pathways suggest that sirtuins contribute to mitochondrial quality control and redox adaptation during aging. However, most mechanistic evidence remains preclinical, and sirtuin effects should be interpreted according to tissue, stressor, and disease context. With advancing age, reduced NAD^+^ availability and altered sirtuin expression likely impair these adaptive responses, contributing to the accumulation of oxidative damage that underlies tissue dysfunction and age-related disease [99].

### 4.5. Sirtuins and Apoptotic Control

Apoptosis maintains tissue homeostasis by eliminating damaged or potentially malignant cells; however, excessive or insufficient apoptosis contributes to age-associated disorders, including neurodegeneration, CVD, cancer, and stem cell exhaustion [1]. Sirtuins regulate apoptosis through chromatin remodeling, p53 and FOXO signaling, mitochondrial integrity, and stress-response pathways. Their effects are highly context-dependent: the same sirtuin may promote survival in non-transformed cells exposed to transient stress, but restrain or facilitate death in malignant cells depending on genetic background and metabolic state [1,23].

Nuclear sirtuins regulate apoptosis partly through epigenetic control of stress-responsive genes. In breast cancer models, SIRT1 modulates histones H3 and H4 acetylation at the promoters of several cancer-related genes, including *AR*, *BRCA1*, *ESR1*, *ESR2*, *EZH2*, and *EP300*, indicating gene-specific transcriptional regulation rather than a uniform pro- or antiapoptotic effect [108]. SIRT6 also affects apoptotic signaling through chromatin-dependent and p53-related mechanisms. In doxorubicin-induced cardiotoxicity models, SIRT6 acted as a corepressor at p53-dependent Fas and FasL target genes, reduced cardiomyocyte apoptosis, and enhanced endogenous antioxidant defense [109]. Conversely, SIRT6 overexpression can induce apoptosis in several cancer cell lines, showing that its effect on cell death depends on cellular context and stress type [110].

The p53 pathway is one of the best-characterized links between sirtuins and apoptosis. Acetylation enhances p53 transcriptional activity toward genes involved in cell-cycle arrest and apoptosis, while SIRT1 deacetylates p53, including Lys382, and can reduce p53-dependent transcriptional and apoptotic responses under sublethal stress conditions [111,112].

This prosurvival function is not absolute: under severe DNA damage, HIPK2-mediated phosphorylation inhibits SIRT1 activity, allowing stronger p53 activation and apoptosis [113]. SIRT7 further promotes cell survival following genomic stress by attenuating DNA damage accumulation, dampening stress-activated p38–JNK signaling, and limiting p53 activation [114]. These findings support a role for SIRT1, SIRT6, and SIRT7 in tuning p53-dependent cell-fate decisions, but they should not be interpreted as uniformly antiapoptotic effects across tissues or cancers. Sirtuins also regulate apoptosis through FOXO transcription factors. SIRT1 deacetylates FOXO1 and FOXO3, and, in selected models, shifts FOXO-dependent transcription toward stress resistance, autophagy, and controlled cell-cycle arrest rather than apoptosis [115]. SIRT2 has a more divergent profile. In MPTP (1-methyl-4-phenyl-1,2,3,6-tetrahydropyridine) models of Parkinson disease, SIRT2 deacetylated FOXO3a, increased Bim expression, and enhanced neuronal apoptosis, whereas SIRT2 deficiency or pharmacologic inhibition reduced this apoptotic response [116]. In contrast, SIRT2 downregulation in HeLa cells induced p53 accumulation through p38 MAPK-dependent mechanisms and promoted apoptosis, suggesting that SIRT2 can either support or limit cell survival depending on the cellular setting [117]. Therefore, SIRT2 should be described as a context-dependent regulator of apoptosis rather than as a uniformly proapoptotic or prosurvival enzyme.

Mitochondrial sirtuins directly influence intrinsic apoptotic signaling. In cardiomyocytes, SIRT3 binds and deacetylates Ku70, enhancing the Ku70–Bax interaction and reducing Bax translocation to mitochondria, cytochrome c release, and activation of caspase-9 and caspase-3 [118]. This mechanism protects cardiomyocytes and other non-transformed tissues under conditions of metabolic, oxidative, or ischemic stress. However, in several cancer models, SIRT3 acts as a tumor suppressor by opposing glycolytic reprogramming, reducing HIF-1α-dependent survival programs, and promoting cell death under defined metabolic conditions [119,120]. SIRT4 also exhibits tissue-specific effects. In H9c2 cardiomyoblasts, SIRT4 attenuated hypoxia-induced apoptosis by reducing caspase-3 and caspase-7 activation, and Bax mitochondrial recruitment [121]. In prostate cancer models, however, SIRT4 promoted apoptosis and inhibited glutamine metabolism, consistent with tumor-suppressive activity [122]. SIRT5 protects cardiomyocytes from oxidative stress-induced apoptosis. Mechanistic data indicate that SIRT5 deacetylates cytochrome c, limits its cytosolic release, and reduces downstream caspase-3 activation [123]. These studies indicate that mitochondrial sirtuins regulate apoptosis through mitochondrial protein deacylation, metabolic control, and redox-sensitive signaling, but their net effects depend on cell type, metabolic state, and disease context.

Because aging is accompanied by NAD^+^ decline, mitochondrial dysfunction, chronic inflammation, and impaired proteostasis, sirtuin-dependent control of apoptosis may become progressively dysregulated with age [92,94,124]. In long-lived tissues such as the heart and nervous system, insufficient sirtuin activity may increase vulnerability to stress-induced cell loss, whereas in cancer, excessive prosurvival sirtuin signaling may help damaged cells evade apoptosis. Thus, therapeutic manipulation of apoptotic sirtuin pathways requires careful attention to tissue specificity, disease stage, and the balance between cytoprotection and tumor suppression.

### 4.6. Role of Sirtuins in Autophagy

Autophagy is a conserved lysosomal degradation pathway that removes long-lived proteins, aggregates, and damaged organelles, thus supporting proteostasis, mitochondrial quality control, and cellular fitness throughout life [125]. Because autophagic efficiency declines in aging phenotypes and age-related diseases, sirtuin-dependent regulation of autophagy provides one mechanism by which NAD^+^-sensitive signaling may influence age-related cellular dysfunction. However, sirtuin effects on autophagy are not uniform and depend on the sirtuin isoform, cell type, metabolic state, and disease context.

SIRT1 is the best-characterized sirtuin in autophagy regulation. Under nutrient limitation or other stresses, SIRT1 physically associates with core autophagy components ATG5, ATG7, and LC3 (ATG8), and promotes their deacetylation, thereby facilitating autophagy induction [126]. In sepsis-induced acute kidney injury models, SIRT1-dependent deacetylation of Beclin-1 at Lys430 and Lys437 promotes autophagosome formation and may also regulate autophagosome-lysosome fusion, indicating that SIRT1 modulates distinct steps of the autophagic pathway rather than functioning as a simple on/off switch [127]. SIRT1 also supports mitophagy in ischemic liver injury. In mouse models, SIRT1 preserved mitochondrial function through an MFN2-dependent mechanism, consistent with improved autophagy-dependent mitochondrial quality control, thereby supporting the selective removal of dysfunctional mitochondria [128]. Apart from direct deacetylation of autophagy proteins, SIRT1 acts through energy-sensing and transcriptional pathways. AMPK and SIRT1 reinforce each other in a positive feedback loop: AMPK increases cellular NAD^+^ and activates SIRT1, while SIRT1 can enhance AMPK activity via upstream kinases and deacetylation of regulatory substrates. This AMPK–SIRT1 axis can inhibit mechanistic target of rapamycin (mTOR) complex 1 and activate unc-51-like kinase 1-dependent autophagy during metabolic stress, while SIRT1-dependent regulation of FOXO proteins and PGC-1α links autophagy and mitophagy to mitochondrial biogenesis and stress resistance [129]. SIRT2 regulates autophagy largely through cytoskeletal and proteostasis-related mechanisms, particularly in neuronal disease models. In Alzheimer disease-related models, SIRT2 activity was associated with α-tubulin deacetylation, impaired microtubule-dependent autophagic trafficking, defective mitochondrial degradation, and reduced clearance of toxic amyloid beta species, whereas SIRT2 inhibition or deletion improved vesicle trafficking and mitochondrial quality control [130]. In Parkinson disease models, SIRT2-mediated deacetylation of α-synuclein at Lys6 and Lys10 promoted α-synuclein aggregation and toxicity. SIRT2 inhibition increased α-synuclein acetylation and reduced its toxic accumulation [131]. These findings indicate that SIRT2 inhibition may restore autophagic or proteostatic flux in selected neurodegenerative settings, although SIRT2 may have different effects in non-neuronal or cancer models.

SIRT3 links mitochondrial metabolism to macroautophagy, chaperone-mediated autophagy, and mitophagy. In adipocytes, SIRT3 overexpression increased autophagic markers and promoted lipid mobilization through AMPK-associated mechanisms [132]. In experimental nonalcoholic fatty liver disease, SIRT3 enhanced BNIP3-dependent mitophagy through ERK–CREB signaling and improved hepatic lipid injury [133]. During *Mycobacterium tuberculosis* infection, SIRT3 supported host defense by coordinating mitochondrial respiration with autophagy activation through PPARα-dependent signaling [134]. These studies support a pro-autophagic or pro-mitophagic role for SIRT3 in defined metabolic and infectious contexts, rather than a universal effect across all tissues.

SIRT4 exerts divergent effects on autophagy depending on disease setting. In doxorubicin-induced cardiotoxicity models, SIRT4 overexpression activated Akt–mTOR signaling, reduced ROS, inhibited excessive autophagy, and attenuated cardiomyocyte apoptosis, thus protecting cells from acute stress [135]. In contrast, in pancreatic ductal adenocarcinoma and some liver cancer models, SIRT4 activated autophagy and inhibited tumorigenesis through a pathway involving suppression of glutamine metabolism, AMPK activation, and p53 phosphorylation [136]. Thus, SIRT4 may suppress maladaptive autophagy in acute cardiac injury while promoting autophagy-dependent tumor suppression in selected cancer models.

SIRT5 regulates autophagy through lysine acylation-dependent control of mitochondrial and metabolic proteins. In brown adipose tissue, SIRT5 deficiency increased protein succinylation, impaired Uncoupling Protein 1 function, reduced mitochondrial respiration, and caused defective mitophagy and thermogenesis dysfunction [137]. Conversely, in colorectal cancer cells, SIRT5 deacetylated lactate dehydrogenase B, enhanced autophagy, and promoted tumorigenic growth [138]. These findings support a bidirectional role for SIRT5 in autophagy, with protective effects on mitochondrial quality control in brown adipose tissue but pro-survival effects in some malignant cells. SIRT6 regulates autophagy mainly through insulin/IGF–Akt–mTOR and FOXO3-related signaling [139]. In isoproterenol-induced cardiac hypertrophy models, SIRT6 activated autophagy, reduced Akt phosphorylation, promoted FOXO3 nuclear retention, increased LC3-II, reduced p62, and attenuated cardiomyocyte hypertrophy [139]. These data support a protective role for SIRT6-mediated autophagy in this cardiac stress model, but this should not be generalized to all forms of cardiac or vascular disease without model-specific evidence [139].

Evidence for SIRT7 in autophagy regulation remains more limited and is highly context-dependent. In human chondrocytes, SIRT7 overexpression increased autophagy markers, reduced oxidative stress, and attenuated degenerative changes, indicating a protective role in cartilage homeostasis [140]. In gastric cancer cells, silencing FOXM1 reduced SIRT7 expression and promoted apoptosis and autophagy through a SIRT7–mTOR–IGF2 signaling axis [141]. In prostate and cervical cancers, SIRT7 depletion suppressed androgen-induced autophagy and proliferation by inhibiting androgen receptor signaling, partly through upregulation of SMAD4 [142]. These findings indicate that SIRT7 can either support or restrain autophagy depending on cell type and oncogenic context.

Overall, sirtuins regulate autophagy through direct deacetylation or deacylation of autophagy proteins, modulation of AMPK-mTOR-ULK1 signaling, control of mitochondrial quality pathways, and transcriptional regulation of stress-response networks. Because individual sirtuins can exert opposing effects in different tissues and disease stages, therapeutic manipulation of sirtuin–autophagy pathways should be considered context-specific rather than uniformly beneficial.

### 4.7. Role of Sirtuins in Cell Proliferation

Cell proliferation supports tissue growth, repair, and regeneration, whereas its dysregulation contributes to neointimal remodeling and tumorigenesis. Sirtuins regulate proliferative capacity through chromatin remodeling, cell-cycle control, metabolic adaptation, autophagy, redox signaling, and oncogenic transcriptional programs. Their effects are strongly context-dependent; the same sirtuin may promote proliferation in one tumor type or vascular setting while restraining growth in another, depending on genetic background, metabolic state, and disease stage [1,23]. SIRT1 has been reported to exert both pro- and antiproliferative effects. In endometrial cancer cells, SIRT1 promoted autophagy-associated proliferation by reducing LC3 acetylation [143]. In contrast, SIRT1 expression was reduced in renal cell carcinoma samples, and SIRT1 overexpression inhibited renal carcinoma cell proliferation, migration, and invasion through AMPK-associated signaling [144]. SIRT1 also suppressed gastric cancer proliferation and metastasis by inhibiting STAT3/MMP-13 signaling, whereas SIRT1 depletion enhanced this pathway [145]. These findings indicate that SIRT1 should be described as a context-dependent regulator of tumor-cell growth rather than as uniformly oncogenic or tumor suppressive.

SIRT2 similarly displays divergent effects on proliferation. In neuroblastoma and pancreatic cancer, N-Myc and c-Myc increased SIRT2 expression, and SIRT2 stabilized Myc oncoproteins, thereby supporting Myc-dependent proliferation; SIRT2 inhibition destabilized Myc and reduced tumor-cell growth [146]. Conversely, SIRT2 has also been identified as a histone deacetylase that suppresses proliferation and migration in neuroblastoma cells, indicating that its effect on growth depends on the dominant substrate and cellular context [147]. Mitochondrial sirtuins connect proliferation to metabolic state. In colorectal cancer cells, SIRT3 deacetylates serine hydroxymethyltransferase 2 at Lys95, thereby stabilizing the enzyme and supporting serine catabolism, NADPH production, cell proliferation, and tumor growth [148]. However, SIRT3 can also act as a tumor suppressor by reducing mitochondrial ROS and limiting HIF-1α-dependent glycolytic reprogramming in cancer models [149,150]. SIRT4 is most often described as a mitochondrial tumor suppressor in the metabolic checkpoint model. After DNA damage, SIRT4 inhibited glutamine catabolism by suppressing glutamate dehydrogenase activity, thereby restricting cell-cycle progression and transformation [87]. Thus, the proliferative effects of SIRT3 and SIRT4 depend on whether mitochondrial metabolism primarily supports biosynthesis, redox balance, or stress-induced growth arrest.

SIRT5 often enhances proliferation in metabolically active cancer cells. Under metabolic stress, SIRT5 desuccinylated serine hydroxymethyltransferase 2 at Lys280, accelerated serine catabolism, and promoted rapid tumor cell growth [151]. Additional studies show that SIRT5 can regulate other metabolic enzymes relevant to tumor growth, but its role outside malignant cells remains less clearly defined and should not be generalized across tissues [47,151]. SIRT6 also has opposing effects on proliferation. In UVB-associated skin carcinogenesis models, SIRT6 increased cyclooxygenase-2 expression by suppressing AMPK signaling, thereby promoting keratinocyte proliferation and survival [152]. In contrast, in hematopoietic stem cells, SIRT6 deletion increased proliferation through aberrant activation of Wnt signaling and impaired long-term self-renewal, indicating that SIRT6 helps preserve stem-cell homeostasis in this context [153]. In multiple myeloma cells, SIRT6 inhibited proliferation and supported genomic stability partly through regulation of ERK-related signaling [154]. These findings support a cell-type-specific role for SIRT6 in growth control rather than a uniform antiproliferative or proproliferative function. Finally, SIRT7 links proliferative signaling to vascular remodeling and androgen receptor-dependent tumor biology. Smooth muscle cell-specific Sirt7 deficiency reduced vascular smooth muscle cell proliferation and attenuated neointimal formation after arterial injury in mice [155]. In prostate cancer, SIRT7 promoted proliferation and androgen receptor signaling. SIRT7 depletion increased SMAD4, reduced androgen receptor signaling, and suppressed xenograft tumor growth [142].

Overall, sirtuins regulate proliferation through several mechanisms, including Myc stabilization, SHMT2-dependent one-carbon metabolism, glutamine metabolic checkpoints, AMPK–COX-2 signaling, Wnt/ERK pathway modulation, and androgen receptor signaling. Because these mechanisms operate differently across normal, injured, and malignant tissues, sirtuin-targeted strategies aimed at proliferation must be evaluated in a disease- and cell type-specific manner.

## 5. Cardiovascular Disease

### 5.1. Metabolic Cardiomyopathy

Metabolic cardiomyopathy refers to myocardial structural and functional impairment driven by systemic metabolic stress, most often obesity, insulin resistance, diabetes, overnutrition/lipotoxicity, dyslipidemia, and related inflammatory or neurohormonal stressors. It is generally used when the myocardial phenotype is not fully explained by dominant ischemic, valvular, infiltrative, genetic, or pressure-overload causes [156,157]. Diabetic cardiomyopathy is among the best-characterized metabolic cardiomyopathies and is often first detected clinically as impaired relaxation and left ventricular diastolic dysfunction. Its development and progression involve overlapping metabolic and cellular abnormalities, including altered substrate utilization, mitochondrial dysfunction, oxidative stress, impaired calcium handling, cardiomyocyte injury/death, hypertrophy, fibrosis, and adverse remodeling [156]. With disease progression, these abnormalities promote cardiomyocyte death, hypertrophy, ventricular dilation, systolic dysfunction, and HF [158].

#### 5.1.1. Clinical Evidence

Current human evidence directly linking sirtuins to metabolic cardiomyopathy remains limited. A rare SIRT1 L107P variant was reported in a family with T1D and ulcerative colitis, and expression of the mutant protein in insulin-producing cells increased inflammatory mediators, supporting a role for SIRT1 in immune-metabolic regulation rather than proving a direct role in DCM [159]. Most evidence for cardiac sirtuin function in metabolic cardiomyopathy is preclinical. Nevertheless, clinical and translational data are beginning to connect metabolic interventions with sirtuin-linked pathways. Human and translational observations also suggest that exercise may interact with the FGF21–SIRT3 axis in diabetic cardiomyopathy, but the mechanistic dependency of the FGF21–β-klotho–AMPK–FOXO3–SIRT3 pathway has been demonstrated mainly in experimental models [160]. Therefore, sirtuin-directed interventions in metabolic cardiomyopathy should be regarded as mechanistically promising rather than clinically validated. No sirtuin-directed intervention has yet been validated clinically for diabetic cardiomyopathy.

#### 5.1.2. Preclinical Evidence

In diabetic and obesity-related cardiomyopathy models, SIRT1 has been implicated mainly in endothelial protection and myocardial NAD^+^-dependent stress adaptation. In endothelial cells, high glucose or advanced glycation end-products reduced SIRT1 activity, increased p53 acetylation, promoted cytochrome-c release, and activated caspase-9 and caspase-3. Pharmacological SIRT1 activation attenuated these apoptotic changes [161]. SIRT1 activation by resveratrol similarly reduced mitochondrial oxidative stress in coronary arterial endothelial cells, although this evidence should be interpreted as pharmacological and cell-model-based rather than proof of clinical efficacy [162]. In the diabetic heart, cardiac overexpression of nicotinamide phosphoribosyltransferase (NAMPT) increased NAD^+^ availability, supported SIRT1-dependent antioxidant signaling, and attenuated high-fat-diet-induced diastolic dysfunction, hypertrophy, fibrosis, apoptosis, and inflammatory signaling [161,163].

SIRT6 has been linked to diabetic vascular and myocardial protection in mechanistic studies. In high-glucose endothelial models and atherosclerosis-prone mice, SIRT6 deacetylated caveolin-1 and promoted its autophagic–lysosomal degradation [164]. This reduced low-density lipoprotein (LDL) transcytosis across the endothelium, thereby limiting atherogenic lipoprotein flux and microvascular dysfunction [164]. In diabetic cardiomyopathy models, SIRT3 deficiency aggravates cardiac dysfunction, mitochondrial injury, oxidative stress, inflammation, necroptosis, and NLRP3 inflammasome activation, whereas preservation or activation of SIRT3 supports mitochondrial homeostasis and attenuates diabetic cardiac injury [165]. SIRT3 is the best-supported mitochondrial sirtuin in diabetic cardiomyopathy models. Global Sirt3 knockout promotes high-fat-diet-induced obesity, insulin resistance, and metabolic syndrome, as well as mitochondrial protein hyperacetylation and impaired oxidative phosphorylation [75]. In cardiac models, SIRT3 deficiency causes mitochondrial cristae disorganization, bioenergetic failure, age-related hypertrophy, fibrosis, and impaired metabolic flexibility. Some mitochondrial defects can be rescued by correcting OPA1 hyperacetylation [166,167]. In DCM models, Sirt3 loss exacerbates cardiac dysfunction by impairing FOXO3a–Parkin-mediated mitophagy, and increasing mitochondrial damage and ROS, whereas SIRT3 overexpression restores mitophagy and improves ventricular performance [168]. SIRT3 also participates in AMPK–FOXO-dependent autophagy and mitophagy during metabolic stress [169]. Exercise appears to act partly through FGF21–β-klotho–AMPK–FOXO3 signaling, which induces Sirt3 expression, reverses diabetes-associated mitochondrial enzyme hyperacetylation, preserves mitochondrial integrity, and improves cardiac function [160]. Beyond mitochondrial quality control, SIRT3 limits profibrotic remodeling. In angiotensin II-induced cardiac fibrosis, SIRT3 deficiency enhances fibroblast-to-myofibroblast transdifferentiation, collagen deposition, NADPH oxidase-derived ROS, and transforming growth factor beta-1 (TGF-β1) signaling, while SIRT3 overexpression attenuates fibrosis partly by inhibiting STAT3–NFATc2 [170]. Overall, these data position SIRT3 as a central preclinical regulator of mitophagy, mitochondrial redox balance, autophagy, and fibrosis in DCM and related metabolic cardiomyopathies, but its therapeutic value in patients remains unproven [171].

SIRT2 may contribute to diabetic endothelial injury [172]. In high-glucose-treated human vascular endothelial cells, SIRT2 is downregulated, whereas SIRT2 overexpression improves cell viability, reduces ROS production, and decreases TNF-α and monocyte chemoattractant protein-1 secretion by limiting acetylation of p53 and NF-κB p65 [172]. This supports a cell-model role in hyperglycemia-induced endothelial dysfunction, but direct in vivo evidence in diabetic cardiomyopathy remains limited.

SIRT4 and SIRT5 have less direct evidence in metabolic cardiomyopathy. SIRT4 regulates leucine catabolism and nutrient-stimulated insulin secretion in pancreatic β-cells [19]. Global Sirt4 knockout mice showed elevated glucose- and leucine-stimulated insulin secretion and developed accelerated age-related insulin resistance, suggesting an indirect cardiometabolic link rather than a proven myocardial mechanism [173]. SIRT5 regulates mitochondrial protein acylation. Sirt5-deficient mice show cardiac mitochondrial protein hypersuccinylation and increased susceptibility to ischemia–reperfusion injury, while SIRT5 overexpression reduces cardiomyocyte death in models of myocardial infarction and ischemia–reperfusion via desuccinylation and stabilization of the trafficking protein TOM1 [174].

SIRT7 supports myocardial stress resistance in aging models, but direct evidence in diabetic cardiomyopathy is limited. Sirt7-deficient mice develop age-dependent cardiac hypertrophy, interstitial fibrosis, and inflammatory cardiomyopathy with collagen accumulation, increased p53 acetylation, basal cardiomyocyte apoptosis, and greater susceptibility to oxidative and genotoxic stress [175]. These data support a role in myocardial homeostasis, not a disease-specific role in diabetic cardiomyopathy [175].

### 5.2. Sepsis-Induced Cardiomyopathy

Beyond DCM, other acquired non-ischemic cardiomyopathic phenotypes with prominent metabolic-mitochondrial stress, including sepsis-induced myocardial dysfunction and alcoholic cardiomyopathy, are closely linked to mitochondrial injury, oxidative stress, and dysregulated inflammatory signaling. Sepsis-induced cardiomyopathy is an acute, often reversible myocardial dysfunction associated with sepsis or septic shock and characterized by impaired contractility, ventricular dilatation in some patients, reduced ejection fraction, mitochondrial dysfunction, inflammatory activation, oxidative stress, and altered substrate utilization [176,177]. No clinical evidence currently demonstrates that sirtuin-targeted therapy improves outcomes in patients with sepsis-induced cardiomyopathy. Available data remain predominantly preclinical and mechanistic.

#### Preclinical Evidence

In lipopolysaccharide-induced cardiac dysfunction, rosmarinic acid improves cardiac function and mitochondrial indices by activating the SIRT1–PGC-1α pathway, and SIRT1 inhibition reduces these effects [178]. Similarly, the adipokine complement C1q tumor necrosis factor-related protein 1 attenuates experimental sepsis-induced cardiomyopathy through SIRT1-dependent pathways that suppress myocardial inflammation, oxidative damage, and apoptosis [179]. Resveratrol reduces ferroptosis and cardiac dysfunction in a rat model through the SIRT1/Nrf2 signaling pathway, with this protection largely reversed by EX-527 [180]. These studies support SIRT1 involvement in experimental sepsis-related myocardial injury, but they do not establish clinical efficacy. SIRT3 also protects septic myocardium in experimental models. SIRT3 activation improves AMPK-related mitochondrial biogenesis, respiration, ATP production, and myocardial injury [181]. More recent work indicates that SIRT3 attenuates sepsis-induced endothelial-to-mesenchymal transition, cardiac remodeling, and fibrosis by promoting PINK1/Parkin-dependent mitophagy [182]. These findings support a model-specific role for SIRT3 in preserving mitochondrial integrity and limiting remodeling during sepsis-induced myocardial injury [180,181].

### 5.3. Alcoholic Cardiomyopathy

Alcoholic cardiomyopathy is an acquired non-ischemic dilated cardiomyopathy caused by prolonged heavy alcohol consumption, and is characterized by ventricular dilatation and systolic dysfunction [183,184].

#### Preclinical Evidence

Among sirtuins, SIRT6 currently has the most direct evidence in alcoholic cardiomyopathy models. In mice exposed to chronic alcohol, myocardial SIRT6 protein declined in parallel with the development of ventricular dilation, systolic dysfunction, mitochondrial fragmentation, oxidative stress, and impaired mitochondrial quality control [185]. Polydatin, a natural stilbene compound structurally related to resveratrol, restores SIRT6 expression, activates AMPK signaling, and normalizes mitochondrial dynamics and mitophagy, reduces ROS production, and improves cardiac function [185]. Sirt6 overexpression mimics these effects, while cardiac Sirt6 knockdown blunts them, supporting SIRT6 dependency in this model. These findings support SIRT6 as a preclinical regulator of mitochondrial integrity and redox homeostasis in alcoholic cardiomyopathy, but not as an established therapeutic target in patients [185]. At a mechanistic level, SIRT6 appears to protect against ethanol-induced mitochondrial injury in experimental alcoholic cardiomyopathy, at least in part through SIRT6–AMPK signaling, modulation of mitochondrial dynamics, enhancement of PINK1–Parkin-dependent mitophagy, reduced oxidative stress, and preservation of mitochondrial function [185]. More broadly, SIRT6-dependent chromatin deacetylation and regulation of transcriptional programs such as NF-κB and NRF2 can suppress inflammatory gene expression and support antioxidant responses [58,186], although these transcriptional mechanisms are less completely defined in the alcoholic heart. Because chronic alcohol exposure can also perturb systemic glucose and lipid metabolism [187], cardiac SIRT6 signaling may act alongside broader metabolic adaptations, supporting SIRT6 as a promising experimental therapeutic target for alcoholic cardiomyopathy and potentially for related toxic or nutritional cardiomyopathic states characterized by mitochondrial injury.

### 5.4. Role of Sirtuins in Myocardial Ischemia–Reperfusion Injury

Myocardial ischemia–reperfusion injury occurs when restoration of coronary blood flow after ischemia paradoxically amplifies cardiomyocyte injury largely through abrupt reoxygenation-associated oxidative stress, calcium overload, mitochondrial dysfunction/mitochondrial permeability transition pore opening, inflammatory activation, and rapid metabolic and ionic shifts [188,189]. Although these mechanisms are clinically relevant after myocardial infarction and reperfusion therapy, direct clinical evidence that pharmacological modulation of sirtuins reduces ischemia–reperfusion injury in patients remains insufficient. Current evidence for sirtuins in this setting is therefore mainly experimental, with limited translational support.

#### Preclinical Evidence

SIRT1 has been implicated in cardiomyocyte survival during ischemia–reperfusion through antioxidant and anti-apoptotic signaling. In mice, cardiac-specific overexpression of SIRT1 reduced infarct size and improved post-ischemic functional recovery, partly through SIRT1, which has been implicated in cardiomyocyte survival during ischemia–reperfusion through antioxidant and anti-apoptotic signaling [190,191]. In diabetic rats, SIRT1 overexpression attenuated diabetes-aggravated myocardial IRI through endothelial nitric oxide synthase (eNOS) activation [192]. SIRT1 has also been linked to Nrf2-dependent antioxidant responses and AMPK-associated autophagic protection in hypoxia-reoxygenation and ischemia–reperfusion models, but these mechanisms are supported mainly by experimental studies and reviews [193]. Exendin-4 increased SIRT1, SIRT3, and AMPK signaling and reduced myocardial injury in a rat ischemia–reperfusion model [194]. Claims about caloric restriction, polyphenols, ginsenosides, or other SIRT1-activating interventions should remain cautious because their cardioprotective effects are pleiotropic and may involve AMPK-dependent as well as AMPK-independent pathways [194]. Excessive SIRT1 expression may also be detrimental in some cardiac contexts; therefore, SIRT1 signaling should be described as dose- and context-dependent rather than uniformly protective [191]. The seminal SIRT1 ischemia–reperfusion study supports protection by cardiac SIRT1 overexpression in mice, but it does not demonstrate clinical efficacy in patients.

SIRT6 protects cardiomyocytes in the ischemia–reperfusion model mainly via AMPK–FOXO3α-dependent antioxidant defense. In cardiomyocytes and in vivo models, Sirt6 overexpression promotes FOXO3α activation, increases antioxidant enzymes such as SOD2 and catalase, reduces ROS, and limits cell death, yielding improved post-ischemic function [103]. Additional experimental work suggests that *Rhaponticum carthamoides* extracts improve myocardial energy metabolism and oxidative stress through SIRT6/Nrf2 signaling, but this should be presented as botanical pharmacology in preclinical myocardial injury rather than clinical preconditioning [195]. In aged hearts, SIRT6 activation reduces charged multivesicular body protein 2B accumulation, restores autophagic flux, and attenuates ischemia–reperfusion vulnerability. Human samples showed an inverse relationship between SIRT6 and charged multivesicular body protein 2B, while mechanistic protection was demonstrated in mice [196].

SIRT3 is the best-supported mitochondrial sirtuin in myocardial IRI. Sirt3 knockdown or deficiency increases susceptibility to simulated and ex vivo IRI, with impaired complex I activity, reduced oxygen consumption, larger infarcts, enhanced mitochondrial permeability transition pore opening, and increased mitochondrial ROS production [197]. Sirt3 knockout in mice impaired coronary microvascular function, caused microvascular rarefaction, and impaired cardiac recovery after myocardial ischemia, indicating that SIRT3 supports both myocardial and coronary microvascular resilience [198]. Following myocardial infarction, Sirt3-deficient mice exhibited more severe ventricular dysfunction and poorer recovery, and these changes were associated with impaired endothelial angiogenic capacity and reduced capillary/pericyte coverage [198]. Mechanistically, SIRT3 deacetylates and activates MnSOD/SOD2, limiting mitochondrial superoxide accumulation during reperfusion and thereby helping to preserve mitochondrial redox homeostasis, respiratory function, and ATP-generating capacity [95,197]. Several interventions further support the SIRT3-centered mitochondrial redox mechanism. Nicotinamide riboside increased cardiac NAD^+^ availability and attenuated experimental myocardial IRI through SIRT3/SOD2 or SIRT3-mitochondrial ROS-JNK signaling [190,199]. Trans-sodium crocetinate reduces myocardial oxidative stress and apoptosis via the SIRT3–FOXO3a–SOD2 pathway, and Sirt3 silencing abolishes this protection [200]. Dexmedetomidine postconditioning similarly limited myocardial IRI in rats by activating the Nrf2–Sirt3–SOD2 axis [201]. In turn, O-GlcNAcylation of SIRT3 at Ser190 during simulated IRI enhanced its deacetylase activity, promoted SOD2 activation, suppressed excessive mitochondrial ROS, and limited maladaptive autophagy, whereas blocking this modification aggravated injury [202]. These findings support SIRT3 as an experimental regulator of mitochondrial redox balance, mitochondrial permeability transition pore sensitivity, and autophagy in reperfused myocardium, without establishing clinical efficacy.

SIRT4 and SIRT5 have been less extensively studied in cardiac IRI, but available evidence supports a protective role in acute experimental injury. In cardiomyocytes and in vivo IRI models, SIRT4 expression decreases after IRI, while forced SIRT4 expression preserved mitochondrial membrane potential, reduced mitochondrial swelling, and decreased levels of cleaved caspase-3 and caspase-9 as well as cardiomyocyte apoptosis, leading to improved ventricular function [203]. Earlier H9c2 cell experiments showed that SIRT4 limits hypoxia-induced apoptosis by reducing Bax translocation to mitochondria and lowering ROS, consistent with an antiapoptotic effect in acute hypoxic stress [121]. However, SIRT4 should not be described as uniformly cardioprotective, because under chronic angiotensin II-induced hypertrophic stress, it inhibits SIRT3-dependent MnSOD activation and promotes hypertrophy [121,204]. Thus, SIRT4 appears protective in acute ischemic or hypoxic injury models but context-dependent in broader cardiac disease.

SIRT5 regulates IRI through mitochondrial desuccinylation and succinate-related metabolism. In Sirt5-null hearts, global mitochondrial protein succinylation is increased, and infarct size after IRI is larger, indicating that endogenous SIRT5 restrains injury susceptibility [205]. Exogenous NAD^+^ reduces oxidative injury and improves post-ischemic function in isolated rat hearts through a SIRT5–succinate dehydrogenase–succinate pathway, implicating SIRT5-dependent control of succinate metabolism in reperfusion ROS generation [206]. More recently, SIRT5 was reported to promote autophagy and reduce cardiomyocyte death in myocardial infarction and ischemia–reperfusion models by desuccinylating and stabilizing TOM1; SIRT5 overexpression increased TOM1, enhanced autophagy, and reduced cell death, whereas TOM1 knockdown reversed these benefits [174]. This mechanism is promising but remains preclinical. Together, these data suggest that SIRT5 protects reperfused myocardium by limiting injurious succinate-related mitochondrial ROS production and facilitating autophagy-mediated clearance of damaged components, although the relative contribution of each mechanism remains model-dependent [174].

SIRT7 shows model- and timing-dependent effects in myocardial injury. SIRT7 resides predominantly in the nucleolus and regulates transcription of nuclear-encoded mitochondrial genes, in part via deacetylation of the transcription factor GABPβ1. Germline Sirt7 deficiency in mice causes spontaneous inflammatory cardiomyopathy with hypertrophy, fibrosis, increased cardiomyocyte apoptosis, and mitochondrial dysfunction, highlighting an essential role in long-term myocardial homeostasis [175]. In acute IRI, however, SIRT7 can act as a target of cardioprotective microRNAs. Bone marrow mesenchymal stem cell-derived exosomes enriched in miR-125b reduce infarct size, improve systolic function, and suppress apoptosis and inflammation in rat IRI models; miR-125b directly targets the 3′-untranslated region of Sirt7, and this short-term SIRT7 downregulation appears necessary for full protection [207]. These findings suggest a temporal model-specific effect: chronic SIRT7 activity supports mitochondrial and contractile integrity, whereas transient repression during acute reperfusion may help dampen stress responses.

### 5.5. Cardiac Hypertrophy and Sirtuin Signaling

Pathological cardiac hypertrophy develops in response to chronic pressure or volume overload and differs from physiological hypertrophy induced by exercise or pregnancy, which is usually reversible and preserves cardiac performance. Maladaptive hypertrophy is accompanied by metabolic remodeling, oxidative stress, fibrosis, and progression toward HF. In this setting, reversible lysine acetylation and acylation regulate transcriptional and mitochondrial pathways, and several sirtuins act as NAD^+^-sensitive modulators of hypertrophic remodeling [208]. Human failing hearts show reduced SIRT6 expression in the study that established SIRT6 as a repressor of IGF-Akt-dependent hypertrophic signaling, but the causal experiments were performed mainly in mouse and cell models [209]. Similarly, studies of SIRT1, SIRT2, SIRT3, SIRT4, SIRT5, and SIRT7 in hypertrophy are dominated by transgenic mice, pressure-overload models, angiotensin II or phenylephrine stimulation, and cultured cardiomyocytes. Therefore, current evidence supports sirtuins as experimental regulators of hypertrophic remodeling, whereas the clinical efficacy of sirtuin-targeted therapy for pathological cardiac hypertrophy has not been established.

#### Preclinical Evidence

Cardiac SIRT1 shows clear dose- and context-dependent effects. In transgenic mice, moderate Sirt1 overexpression protected the aging heart from dilation, apoptosis, and oxidative stress, whereas higher overexpression induced spontaneous hypertrophy, oxidative stress, and systolic dysfunction, indicating a non-linear response rather than uniformly protective signaling [210]. Under hemodynamic load, SIRT1 can support mitochondrial adaptation but may also promote maladaptive remodeling depending on metabolic context. Long noncoding RNA MHRT attenuated experimental cardiac hypertrophy by promoting SIRT1 SUMOylation and activation of the SIRT1-PGC-1α-PPARα pathway, whereas in pressure-overloaded hearts, a PPARα-SIRT1 complex suppressed estrogen-related receptor-dependent mitochondrial gene programs; haploinsufficiency of either Pparα or Sirt1 blunted hypertrophy, while their simultaneous upregulation aggravated dysfunction [211,212]. Consistent with this duality, cardiomyocyte-specific Sirt1 deletion sensitized male mice to pressure overload in one model, whereas another pressure-overload study reported that SIRT1 aggravated hypertrophic HF by shifting energy metabolism [213]. Thus, SIRT1 should be described as model-, dose-, and metabolic-state dependent rather than simply antihypertrophic.

SIRT3 is the best-supported mitochondrial sirtuin opposing pathological cardiac hypertrophy. *Sirt3*-null mice are born with structurally normal hearts but develop age-dependent hypertrophy, interstitial fibrosis, and contractile dysfunction, and they show exaggerated responses to pressure overload or neurohormonal stimulation [102]. Mechanistically, SIRT3 deacetylates and activates FOXO3a, increasing MnSOD/SOD2 and catalase expression, lowering mitochondrial ROS, and suppressing ROS-sensitive Ras–MAPK and PI3K–Akt signaling [102]. In cardiac hypertrophy models, SIRT3 protects against pathological remodeling by preserving mitochondrial function and limiting oxidative stress, with broader evidence indicating that SIRT3 also supports mitochondrial fatty-acid oxidation through deacetylation and activation of long-chain acyl-CoA dehydrogenase [79,102]. Additional experimental work shows that SIRT3 can reduce poly(ADP-ribose) polymerase 1 acetylation/activity and help preserve NAD^+^ during hypertrophic stress [214]. CD38-mediated NAD^+^ depletion enhances angiotensin II-induced hypertrophy through inhibition of the SIRT3–FOXO3 pathway and activation of Ca^2+^–calcineurin–NFAT signaling, further supporting the importance of the NAD^+^–SIRT3 axis in pathological cardiac growth [215]. SIRT6 acts primarily through chromatin-dependent repression of hypertrophic transcription. Cardiac Sirt6 deficiency causes hypertrophy and HF, whereas SIRT6 transgenesis protects mice from hypertrophic stimuli. SIRT6 interacts with c-Jun at promoters of IGF-Akt pathway genes and deacetylates histone H3 lysine 9 (H3K9), thereby repressing IGF-PI3K-Akt signaling [209]. Additional experimental studies show that SIRT6 suppresses cardiomyocyte hypertrophy by inhibiting NF-κB-dependent transcription, promoting p300 degradation, suppressing STAT3 and NFATc4 signaling, and regulating autophagy through FOXO3-dependent pathways; these mechanisms should be presented as model-specific rather than universal [139,216,217]. SIRT6 also attenuated angiotensin II-mediated myocardial hypertrophy, dysfunction, and fibrosis through AMPK–ACE2 signaling in rodents, linking SIRT6 to the renin–angiotensin system counter-regulation in experimental remodeling [218]. SIRT2 has conflicting reported effects in cardiac hypertrophy and should be framed cautiously. In one major preclinical study, SIRT2 expression decreased in hypertrophic mouse hearts. Sirt2 knockout exacerbated aging- and angiotensin II-induced hypertrophy, fibrosis, and systolic dysfunction, whereas cardiomyocyte-specific SIRT2 overexpression reduced remodeling [219]. Mechanistically, SIRT2 deacetylated LKB1, enhanced AMPK activation, and contributed to the antihypertrophic effect of metformin [219]. A second study showed that SIRT2 binds and deacetylates NFATc2, limiting NFAT nuclear localization and transcriptional activation; SIRT2 loss sustained NFAT signaling and fetal-gene induction [220]. Endothelial progenitor cell-derived exosomal circ_0018553 was also reported to protect cardiomyocytes from angiotensin II-induced hypertrophy through the miR-4731/SIRT2 axis [221]. These data support an antihypertrophic role for SIRT2 in selected preclinical models, but clinical evidence is lacking. Among mitochondrial isoforms, SIRT4 generally favors pathological remodeling in angiotensin II-induced hypertrophy. In murine models, cardiac Sirt4 is upregulated during angiotensin II-induced stress. Sirt4 transgenic mice develop more severe hypertrophy and reduced cardiac function, while Sirt4 deficiency attenuates hypertrophic growth and fibrosis [204]. Mechanistically, SIRT4 disrupts SIRT3 binding to MnSOD, increases MnSOD acetylation, reduces MnSOD activity, and increases mitochondrial ROS, thereby amplifying hypertrophic and profibrotic signaling [204]. This chronic hypertrophic phenotype contrasts with the antiapoptotic role of SIRT4 reported in acute hypoxic or ischemic injury models, so its cardiac function should be described as stress- and disease-stage dependent. SIRT5 supports mitochondrial metabolism under cardiac stress, but published models are not fully concordant. Systemic Sirt5-null mice subjected to transverse aortic constriction show impaired oxidative metabolism, accelerated cardiac dysfunction, and increased mortality, supporting a role for SIRT5 in maintaining mitochondrial energy production during pressure overload [222,223]. In contrast, postnatal cardiomyocyte-specific Sirt5 ablation produced persistent protein hypersuccinylation but normal survival after chronic pressure overload, indicating that the impact of SIRT5 loss depends on knockout strategy, developmental timing, and systemic versus cardiac-specific deletion [222]. SIRT5 also regulates long-chain fatty-acid oxidation. Metabolomics-assisted proteomics identified ECHA/HADHA, the α-subunit of the mitochondrial trifunctional β-oxidation enzyme, as a major cardiac SIRT5 substrate. In Sirt5-deficient mice, ECHA hypersuccinylation reduced its activity and was associated with impaired fatty-acid metabolism, decreased ATP production, and hypertrophic cardiac remodeling, and contributed to hypertrophic cardiomyopathy under metabolic stress [224]. Recent research further suggests that the flavonoid nobiletin prevents pressure-overload HF through SIRT5-dependent desuccinylation of p300 at lysine 1568 and inhibition of p300 histone acetyltransferase activity; this mechanism is promising but very recent and should be interpreted as preclinical [225]. Together, these data indicate that SIRT5 regulates cardiac protein succinylation and supports oxidative metabolism during hypertrophic stress, but its effect on pressure-overload outcomes depends on the experimental model, timing of Sirt5 loss, and systemic versus cardiomyocyte-restricted deletion [222]. SIRT7 also exerts context-dependent antihypertrophic effects. Germline Sirt7-deficient mice have reduced lifespan and develop cardiac hypertrophy, extensive fibrosis, inflammatory cardiomyopathy, and increased cardiomyocyte stress susceptibility [175]. Cardiomyocyte-specific studies show that SIRT7 interacts with the transcription factor GATA4 and promotes its deacetylation [226]. Loss of Sirt7 enhances GATA4 acetylation, augments phenylephrine- and pressure-overload-induced hypertrophy, and worsens systolic dysfunction, whereas restoring SIRT7 activity or GATA4 reduction mitigates these effects [226]. Nicotinamide mononucleotide attenuated agonist-induced hypertrophy in a SIRT7-dependent manner in vitro, linking SIRT7’s antihypertrophic effects to myocardial NAD^+^ availability [226]. These data support a protective role for cardiomyocyte SIRT7 in stress-induced hypertrophy, while other SIRT7 effects in fibrosis and tissue repair should be considered separately because they may differ by cell type and injury phase.

### 5.6. Role of Sirtuins in Cardiac Fibrosis

Cardiac fibrosis is a major component of maladaptive ventricular remodeling and results from sustained activation of cardiac fibroblasts into alpha-smooth muscle actin-positive myofibroblasts, excessive deposition of collagen and other extracellular matrix proteins, and progressive ventricular stiffening. TGF-β-Smad signaling, angiotensin II, oxidative stress, and inflammatory cues are established drivers of fibroblast activation and fibrotic remodeling; several sirtuins modulate these pathways in experimental models [227]. Direct clinical evidence that sirtuin-targeted treatment reduces cardiac fibrosis in patients is not available. The current literature consists mainly of rodent pressure-overload, angiotensin II, dilated cardiomyopathy, myocardial infarction, and cultured cardiac fibroblast models, with review-level translational discussion but no established antifibrotic sirtuin therapy [227,228]. Thus, SIRT1, SIRT2, SIRT3, SIRT5, and SIRT6 can be described as experimental antifibrotic regulators in defined models, whereas SIRT4 and SIRT7 show more context-dependent behavior.

#### Preclinical Evidence

SIRT1 has been linked to antifibrotic effects through Smad deacetylation. In a rat model of dilated cardiomyopathy, resveratrol improved cardiac function, reduced myocardial fibrosis, increased SIRT1 expression, and decreased Smad3 acetylation. The authors concluded that resveratrol attenuated myocardial fibrosis through the SIRT1/Smad3 deacetylation pathway. This evidence is experimental and does not exclude SIRT1-independent actions of resveratrol [229]. SIRT1 activation has also been reported to attenuate fibrosis in a rodent pressure-overload model by modifying Smad2/3 transactivation, supporting a Smad-related antifibrotic mechanism in pressure overload [230]. SIRT3 attenuates fibrotic remodeling mainly by limiting oxidative stress in fibroblasts and pressure-overloaded myocardium. In angiotensin II-stimulated cardiac fibroblasts, the hydrogen sulfide donor NaHS increased SIRT3 expression and reduced fibroblast proliferation, collagen-related markers, mitochondrial dysfunction, and oxidative stress. Sirt3 silencing weakened these effects. In transverse aortic constriction (TAC), NaHS reduced myocardial fibrosis in wild-type mice but not in Sirt3-deficient mice, supporting a SIRT3-dependent antifibrotic mechanism in this model [227,231].

SIRT6 has antifibrotic effects in angiotensin II-mediated remodeling and in broader fibrosis models, but the mechanisms should not be merged. In angiotensin II-treated mice and cardiac cells, SIRT6 attenuated myocardial fibrosis and injury through AMPK–ACE2 activation and suppression of CTGF–FKN signaling [218]. Separately, SIRT6 deficiency was shown to transcriptionally upregulate TGF-β signaling and induce fibrosis in mice, with SIRT6-deficient fibroblasts undergoing spontaneous myofibroblast transformation through hyperactivation of TGF-β signaling [232]. Therefore, SIRT6 can be described as an experimental antifibrotic regulator, but the cardiac angiotensin II study should be cited for AMPK–ACE2/CTGF–FKN signaling, not for H3K9 deacetylation at Smad target promoters unless that exact mechanism is supported by a separate primary source.

SIRT2 limits fibrosis in selected angiotensin II- and aging-related remodeling models. In mice, Sirt2 deficiency aggravated interstitial and perivascular fibrosis, hypertrophy, and systolic dysfunction, whereas cardiomyocyte-specific SIRT2 overexpression reduced remodeling. Mechanistically, SIRT2 deacetylated liver kinase B1, promoted AMPK activation, reduced oxidative stress, and contributed to metformin-associated cardioprotection [219]. These findings support an antifibrotic role for SIRT2 in this experimental context, but not a general antifibrotic effect across all fibrosis models.

SIRT5 regulates fibrotic remodeling under pressure overload, but conclusions depend on the genetic model. Whole-body Sirt5-null mice exposed to TAC show impaired oxidative metabolism and increased mortality, supporting a role for SIRT5 in adaptation to pressure overload [223,233]. In contrast, postnatal cardiomyocyte-specific Sirt5 ablation causes persistent cardiac protein hypersuccinylation but normal survival after chronic pressure overload, indicating that the severe phenotype of global Sirt5 deficiency may reflect developmental or extracardiac effects. SIRT5 overexpression preserved cardiac function after TAC, reduced left ventricular dilation and ejection-fraction impairment, and suppressed transcriptional programs related to glycolytic shift, immune activation, and fibrotic signaling. Recent work indicates that nobiletin prevents pressure-overload heart failure through SIRT5-dependent inhibition of p300 acetyltransferase activity, including p300 desuccinylation at lysine 1568, but this remains preclinical pharmacology rather than established antifibrotic therapy [225].

SIRT4 generally promotes fibrotic remodeling under chronic angiotensin II stress. Cardiomyocyte-specific Sirt4 overexpression aggravates angiotensin II-induced hypertrophy, fibrosis, and cardiac dysfunction, whereas Sirt4 deficiency attenuates these responses. Mechanistically, SIRT4 increases mitochondrial ROS by interfering with the SIRT3–MnSOD axis, thereby amplifying redox-sensitive pathological remodeling [204]. This statement should remain restricted to chronic pathological stimulation, because SIRT4 has different effects in acute hypoxic or ischemic injury models [204].

SIRT7 has context-dependent effects in cardiac fibrosis and repair. In primary cardiac fibroblasts, angiotensin II increases SIRT7 expression and phosphorylation. *Sirt7* knockdown reduced fibroblast proliferation, ECM deposition, α-SMA expression, and focal adhesion formation, whereas Sirt7 overexpression enhanced angiotensin II-induced myofibroblast differentiation. SIRT7 also promoted Smad2 and ERK phosphorylation in this fibroblast model [234]. Conversely, germline Sirt7-deficient mice develop cardiac hypertrophy, inflammatory cardiomyopathy, extensive fibrosis, increased collagen III accumulation, p53 hyperacetylation, and increased myocardial apoptosis, indicating that chronic systemic SIRT7 loss impairs myocardial homeostasis [175]. After myocardial infarction, SIRT7 maintains transforming growth factor-β receptor I through autophagy-related regulation and contributes to tissue repair, showing that SIRT7 may promote reparative fibrosis in infarct healing while also supporting long-term myocardial integrity [235]. SIRT7 should therefore not be classified simply as antifibrotic or profibrotic; its effect depends on cell type, injury phase, and experimental model.

### 5.7. Role of Sirtuins in Heart Failure

Heart failure (HF) is the end stage of many cardiovascular disorders and affects more than 60 million people worldwide. It is characterized by contractile dysfunction, impaired energy metabolism, oxidative stress, adverse remodeling, and progressive cardiomyocyte loss. Sirtuins couple NAD^+^ availability and metabolic state to stress-responsive transcriptional, mitochondrial, inflammatory, and survival pathways in cardiomyocytes, endothelial cells, and fibroblasts. Human myocardial studies, animal models, and cell-based experiments most consistently implicate SIRT1, SIRT3, and SIRT6 in HF-related remodeling, whereas evidence for SIRT2, SIRT4, SIRT5, and SIRT7 remains more model-specific [236]. Direct clinical evidence that sirtuin-targeted treatment improves HF outcomes in patients remains lacking.

#### 5.7.1. Clinical and Human Evidence

In atrial myocardium from patients with advanced HF, SIRT1 protein was reduced compared with donor hearts, together with lower MnSOD, thioredoxin-1, and Bcl-xL, higher Bax and acetylated p53, reduced nuclear FOXO1, and increased oxidative damage and apoptosis [237]. These findings support an association between reduced SIRT1 and pro-oxidant/proapoptotic remodeling in end-stage HF, but they do not prove that SIRT1 loss independently causes HF progression [237]. SIRT6 expression has been reported to decrease in failing human hearts, and experimental data show that SIRT6 restrains cardiac hypertrophy by repressing insulin-like growth factor (IGF)-Akt pathway genes through c-Jun-dependent recruitment and histone H3 lysine 9 deacetylation [214]. Direct human HF evidence for SIRT2, SIRT3, SIRT4, SIRT5, and SIRT7 remains limited; most mechanistic data for these isoforms come from animal or cell models.

#### 5.7.2. Preclinical and Mechanistic Evidence

SIRT1 generally protects the stressed myocardium when maintained within a physiological range. In experimental myocardial injury and HF models, SIRT1 supports mitochondrial biogenesis, respiratory function, and energy production partly through PGC-1α. The RNA-binding protein La ribonucleoprotein 7 preserved SIRT1 stability and deacetylase activity in experimental HF, maintaining PGC-1α-dependent mitochondrial biogenesis, oxidative phosphorylation, ATP production, and cardiac function [238]. Long noncoding RNA XIST protected hypoxia-injured H9c2 cells through the miR-486-5p/SIRT1 axis, but this evidence is preclinical and should not be presented as proof of benefit in chronic human HF [238,239]. SIRT1 is dose-dependent: moderate activity is generally cardioprotective, whereas excessive cardiac SIRT1 signaling can aggravate hypertrophic dysfunction, so indiscriminate activation should not be assumed to be beneficial [213,240].

SIRT3 is the best-supported mitochondrial sirtuin in HF models. Experimental SIRT3 deficiency increases mitochondrial protein hyperacetylation, weakens oxidative metabolism, raises oxidative stress, and increases susceptibility to aging- or pressure-overload-related cardiac dysfunction [167,241]. Endothelial SIRT3 also contributes to HF adaptation. Endothelial-specific Sirt3 deletion disrupts apelin-dependent glucose transport from coronary microvascular endothelial cells to cardiomyocytes, reduces cardiomyocyte glucose utilization, impairs coronary flow, and worsens pressure-overload HF in mice [242]. These findings support SIRT3 as a regulator of both cardiomyocyte mitochondrial resilience and coronary microvascular metabolic support.

SIRT6 is reduced in failing human hearts and in experimental pressure overload models [64]. SIRT6 protects the heart mainly through chromatin-dependent repression of hypertrophic and profibrotic pathways. Cardiac SIRT6 suppresses IGF–PI3K–Akt signaling by interacting with c-Jun and deacetylating H3K9 at IGF-pathway promoters. Sirt6-deficient mice develop cardiac hypertrophy and HF, whereas SIRT6 transgenic expression protects against hypertrophic stimuli [107,209,232]. SIRT6 deficiency also promotes fibroblast-to-myofibroblast conversion through hyperactivation of transforming growth factor beta (TGF-β) signaling, supporting an antifibrotic role for SIRT6 in experimental fibrosis [232]. In angiotensin II-mediated myocardial injury, SIRT6 activates AMPK–ACE2 signaling and suppresses connective tissue growth factor (CTGF)–fractalkine (FKN) signaling, thereby reducing myocardial fibrosis and injury; this mechanism should be restricted to angiotensin II remodeling models and not generalized to all HF phenotypes [218,232]. Xanthenone has been reported to improve post-infarction remodeling in rodents through the miR-122/SIRT6/ACE2 axis, but this remains preclinical pharmacological evidence [218,243].

Evidence for SIRT2 in HF is indirect and comes mainly from hypertrophy and remodeling models. One study showed that SIRT2 restrains aging- and angiotensin II-induced hypertrophic remodeling by deacetylating liver kinase B1 (LKB1), activating AMP-activated protein kinase (AMPK), and preserving the antihypertrophic response to metformin [244]. A related mechanism showed that SIRT2 deacetylates nuclear factor of activated T cells c2 (NFATc2), limiting its nuclear localization and transcriptional activity [220]. In contrast, another study found that Sirt2 deletion or pharmacological SIRT2 inhibition improved cardiac function after pressure overload and ischemia–reperfusion injury by preserving nuclear factor erythroid 2-related factor 2 (Nrf2)-dependent antioxidant signaling [245]. Therefore, SIRT2 should not be described as uniformly cardioprotective or uniformly harmful.

SIRT4 has divergent effects depending on the model. In H9c2 cardiomyoblasts, SIRT4 overexpression limits hypoxia-induced apoptosis by reducing Bax translocation and caspase-3 and caspase-9 activation, whereas knockdown has the opposite effect. This supports an antiapoptotic role only in an acute in vitro hypoxic-stress model [121]. Whether this antiapoptotic action translates into chronic HF models in vivo has not yet been established. In contrast, SIRT4 promotes angiotensin II-induced pathological hypertrophy, fibrosis, and cardiac dysfunction by increasing mitochondrial reactive oxygen species and inhibiting the SIRT3–MnSOD antioxidant axis [204]. More recent pressure-overload data also support a maladaptive role for SIRT4 in HF development through reactive oxygen species-mediated profibrotic signaling [246].

SIRT5 regulates mitochondrial desuccinylation and oxidative metabolism during cardiac stress. Sirt5-deficient mice subjected to pressure overload show impaired cardiac oxidative metabolism and reduced survival, indicating that SIRT5 is required for adaptation to hemodynamic stress [233]. SIRT5 overexpression preserves cardiac function and reduces fibrosis-related remodeling after transverse aortic constriction, supporting a protective effect in experimental pressure overload [223]. In a pressure-overload HF model, quercetin improved left ventricular function and reduced fibrosis at least partly by enhancing SIRT5-dependent desuccinylation and activation of IDH2, thereby reinforcing mitochondrial antioxidant capacity [247]. This mechanism remains experimental.

SIRT7 has cell-type- and disease-stage-dependent effects in myocardial remodeling. Germline Sirt7-deficient mice show reduced lifespan, cardiac hypertrophy, inflammatory cardiomyopathy, increased cardiomyocyte apoptosis, and mitochondrial dysfunction, indicating that chronic SIRT7 loss disrupts myocardial homeostasis [175,235]. In cardiac fibroblasts, angiotensin II increases SIRT7 abundance and phosphorylation, and SIRT7 promotes profibrotic differentiation through Smad2 and ERK signaling. After myocardial infarction, SIRT7 contributes to tissue repair by maintaining TGF-β receptor I expression and TGF-β signaling, suggesting that SIRT7 may support scar formation and remodeling after acute injury [234,235]. However, the precise role of SIRT7 in chronic HF remains incompletely defined and may differ between cardiomyocytes and fibroblasts.

Overall, sirtuins regulate several processes relevant to HF progression, including mitochondrial energetics, redox balance, cardiomyocyte survival, inflammatory signaling, and fibroblast activation. SIRT1, SIRT3, and SIRT6 have the strongest evidence for cardioprotective activity when maintained at physiological or moderately enhanced levels, whereas SIRT2, SIRT4, SIRT5, and SIRT7 require more cautious wording because their effects vary by isoform, cell type, disease model, and stress duration.

### 5.8. Role of Sirtuins in Atherosclerosis

Atherosclerosis is a chronic inflammatory disease of the arterial wall driven by lipid accumulation, endothelial dysfunction, immune-cell infiltration, vascular smooth muscle cell (VSMC) remodeling, plaque formation, and, in advanced disease, thrombotic complications [248]. Sirtuins regulate several of these processes through redox-, nutrient-, and NAD^+^-sensitive deacylase activity, but their effects are isoform-, cell-type-, and model-dependent [249].

#### 5.8.1. Clinical and Human Evidence

SIRT1 expression is reduced in human atherosclerotic plaques, especially in VSMCs, and lower SIRT1 is associated with impaired DNA-damage responses, VSMC senescence or apoptosis, and features of plaque progression [250]. This supports an association in human tissue, whereas causality is mainly supported by experimental models. Serum SIRT6 concentrations have been reported to be lower in patients with stable angina or acute coronary syndrome, but this is a coronary artery disease (CAD)-associated biomarker finding and should not be presented as proof that reduced SIRT6 causes atherosclerosis [251,252]. Additional human monocyte data indicate that reduced SIRT1 is accompanied by increased acetylated p53, oxidative stress, low-density lipoprotein receptor-1 expression, NF-κB activation, and inflammatory gene expression, supporting an association between SIRT1 repression and proinflammatory myeloid-cell activation in CAD [253]. Human genetic studies also suggest that sirtuin variation may influence coronary atherosclerosis: SIRT6 tagSNPs rs352493 and rs3760908 were associated with angiographic CAD severity in a Chinese Han population, whereas recent case-control data linked selected SIRT3 polymorphisms with CAD susceptibility and circulating malondialdehyde, a marker of lipid peroxidation [254,255]. These findings are associative and should not be presented as proof that altered SIRT1, SIRT3, or SIRT6 activity causes human atherosclerosis.

#### 5.8.2. Preclinical and Mechanistic Evidence

Endothelial SIRT1 protects against atherosclerotic lesion formation in ApoE-deficient mice. Endothelial-specific Sirt1 overexpression reduces aortic lesion area without major changes in plasma lipids, and this protection is linked to improved endothelial nitric oxide synthase (eNOS) activity, increased nitric oxide bioavailability, and reduced endothelial apoptosis [256]. SIRT1 also limits endothelial activation and thrombosis. Pharmacological or genetic inhibition of SIRT1 increases endothelial tissue factor expression through NF-κB p65 activation and accelerates arterial thrombus formation in vivo [257]. In VSMCs, SIRT1 preserves genome integrity by regulating the DNA repair protein Nijmegen breakage syndrome 1 (NBS1). Experimental SIRT1 loss impairs DNA repair and promotes DNA-damage-associated VSMC senescence or apoptosis, mechanisms also observed in human plaques [250,258]. In macrophage-related models, partial Sirt1 deficiency increases oxLDL uptake and foam-cell formation, whereas SIRT1 activation reduces LOX-1-mediated lipid uptake and suppresses NF-κB-dependent inflammatory signaling [259]. The mTOR–SIRT1 axis also regulates macrophage lipid handling. mTOR activation promotes foam-cell formation and reduces foam-cell egress by suppressing SIRT1 signaling [260]. miR-217 is upregulated in atherosclerosis-related models and oxLDL-treated macrophages, directly targets SIRT1, and promotes inflammatory responses. miR-217 inhibition restores SIRT1 expression and reduces inflammatory activation and lesion burden in experimental models [261].

SIRT6 regulates atherosclerosis through distinct hepatic, macrophage, VSMC, and endothelial mechanisms that should not be merged into one pathway. In lipid metabolism, SIRT6 can repress PCSK9 transcription through FoxO3-dependent recruitment to the Pcsk9 promoter and H3K9/H3K56 deacetylation, thereby reducing circulating LDL cholesterol in high-fat-diet-fed mice. This is a hepatic cholesterol-regulatory mechanism rather than direct proof of plaque-cell protection [262,263]. Within atherosclerotic plaques, SIRT6 promotes macrophage autophagy and has been reported to improve plaque stability, whereas Sirt6 deficiency is associated with larger and more vulnerable plaques with impaired autophagy [264]. In endothelial cells, SIRT6 limits adhesion molecule expression, inflammatory activation, and pyroptosis through the Lin28B/let-7 pathway. This remains experimental and should be treated as model-specific rather than established in human plaques [265].

SIRT7 evidence in atherosclerosis is mainly preclinical and VSMC-focused. A p53-dependent lincRNA-p21–miR-17-5p–SIRT7 axis has been reported in human samples, ApoE-deficient mice, and VSMC models. The lincRNA-p21 sponges miR-17-5p, thereby increasing SIRT7 expression. Elevated SIRT7 suppresses pathological VSMC proliferation and migration, promotes apoptosis of hyperproliferative VSMCs, and modulates Wnt–β-catenin signaling to reduce neointimal formation and plaque vulnerability [153]. Additionally, SNHG7-003 suppresses oxLDL-induced VSMC proliferation, migration, and invasion through the miR-1306-5p/SIRT7 axis, but this finding is based on VSMC models and should not be generalized to all plaque compartments [266].

Mitochondrial sirtuins regulate vascular oxidative stress and metabolic flexibility, but their effects on plaque burden are not uniform [249]. SIRT3 deacetylates and activates SOD2, limiting mitochondrial ROS accumulation in vascular disease models and protecting against endothelial dysfunction, hypertension, and metabolic stress [96,267]. In experimental atherosclerosis, Sirt3 impairment and SOD2 hyperacetylation have been linked to vascular oxidative stress and apoptosis [96,268]. However, Sirt3 deletion does not consistently increase atherosclerotic lesion size in LDLR-deficient mice, indicating that SIRT3 may influence systemic oxidative stress, metabolic adaptation, endothelial function, or plaque vulnerability more clearly than lesion burden itself [269]. SIRT4 evidence is limited to endothelial injury models. In oxLDL-treated human umbilical vein endothelial cells, SIRT4 expression decreases, whereas SIRT4 overexpression enhances cell survival, reduces apoptosis, and suppresses PI3K–Akt–NF-κB signaling and the expression of proinflammatory cytokines IL-1β, IL-6, and TNF-α [270]. This supports a possible anti-inflammatory endothelial role in vitro, not a proven antiatherosclerotic effect in vivo [270].

SIRT5 should be discussed mainly in the context of atherothrombosis rather than plaque formation. In mouse models, Sirt5 overexpression promotes arterial thrombosis by increasing endothelial plasminogen activator inhibitor-1 expression and inhibiting fibrinolysis, while SIRT5 silencing in human aortic endothelial cells reduces this factor (as well as proinflammatory mediators) via AMPK activation and ERK1/2 inhibition [271]. Therefore, SIRT5 may connect mitochondrial metabolism, endothelial inflammation, and thrombotic risk, but current evidence does not establish SIRT5 as a direct regulator of atherosclerotic plaque size.

SIRT2 has preclinical antiatherogenic evidence through macrophage and oxidative-stress pathways. In LDLR-deficient mice, pharmacological or genetic SIRT2 activation reduces plaque area and increases markers of plaque stability by shifting macrophage polarization away from a proinflammatory M1 phenotype [272]. In hypertensive atherosclerotic mice, miR-140-5p aggravates hypertension and oxidative stress by targeting Nrf2 and SIRT2, whereas miR-140-5p inhibition restores Nrf2–SIRT2 signaling and attenuates oxidative vascular injury [273].

Overall, the strongest atherosclerosis evidence supports protective roles for SIRT1 and SIRT6 in endothelial function, VSMC stress responses, macrophage lipid handling, inflammation, autophagy, and plaque stability. SIRT2, SIRT3, SIRT4, SIRT5, and SIRT7 should be presented more cautiously because their evidence is mainly preclinical, cell-type specific, or related to risk-factor biology and atherothrombosis rather than direct human plaque progression.

### 5.9. Role of Sirtuins in Coronary Artery Disease

CAD is the clinical manifestation of progressive atherosclerotic plaque formation in the epicardial coronary arteries and remains a leading cause of morbidity and mortality worldwide [274]. In CAD, sirtuins influence vascular and myocardial biology through regulation of oxidative stress, inflammatory signaling, endothelial function, apoptosis, mitochondrial metabolism, and vascular smooth muscle cell (VSMC) behavior [259]. The strongest human evidence concerns SIRT1, whereas data for SIRT3 and SIRT6 are mainly genetic, associative, or extrapolated from vascular and metabolic models [254,275].

#### 5.9.1. Clinical and Human Evidence

Human data are strongest for SIRT1. Peripheral blood monocytes from patients with stable CAD or acute coronary syndrome display significantly reduced SIRT1 mRNA and protein levels compared with healthy controls [252,275]. In the same study, high-density lipoprotein (HDL) from healthy subjects induced SIRT1 expression in THP-1 monocytes more effectively than HDL from patients with stable CAD or acute coronary syndrome, and this impaired effect was attributed to reduced HDL-associated paraoxonase-1 activity [252]. These findings support an association between CAD, impaired HDL function, and reduced monocytic SIRT1 expression, but they do not establish reduced SIRT1 as the initiating cause of CAD. Reduced monocytic SIRT1 expression in CAD is associated with increased acetylation of p53, enhanced oxidative stress, lectin-like oxidized low-density lipoprotein receptor-1, NF-κB activation, and a proinflammatory phenotype [253]. These findings support an association between reduced SIRT1 signaling and proinflammatory myeloid activation in CAD, but they do not prove that SIRT1 loss alone causes coronary plaque progression.

Several human studies have examined SIRT1 polymorphisms in CAD or acute coronary syndrome. SIRT1 rs4746720, rs12413112, and rs1467568 were associated with CAD risk in an Iranian case-control study, whereas rs3758391 was associated with acute coronary syndrome and SIRT1 mRNA expression in a Chinese cohort [275,276]. The rs3758391 promoter variant lies within a p53-binding region and affects nutrient-sensitive SIRT1 transcription [277]. In South African Indians with early-onset CAD, rs1467568 and rs7895833 were examined, but the study did not identify a clear CAD-control association for these variants [278]. Therefore, SIRT1 polymorphisms should be described as population-dependent susceptibility signals rather than universal CAD predictors. The miR-34a–SIRT1 axis has also been investigated in human CAD. In endothelial progenitor cells from patients with CAD, miR-34a expression was higher, and SIRT1 protein expression was lower than in non-CAD controls, and functional experiments showed that miR-34a can regulate SIRT1 expression [279]. Statin treatment was reported to modify this miR-34a–SIRT1 relationship, supporting a regulatory association and experimental responsiveness, but not proving that miR-34a-mediated SIRT1 repression alone determines CAD progression. The long noncoding RNA (lncRNA) CAMK2D-associated transcript 1 (C2dat1) is upregulated in coronary artery segments from CAD patients and in VSMCs exposed to proliferative or inflammatory stimuli compared with normal arterial tissues [280]. This human tissue finding supports an association between C2dat1 expression and CAD, but it does not prove that C2dat1 drives coronary plaque progression in patients.

Human evidence for SIRT3 and SIRT6 is more limited and mainly genetic or associative. Rare or low-frequency SIRT3 variants and promoter polymorphisms have been associated with myocardial infarction or CAD susceptibility [281]. Several variants altered promoter activity, supporting a possible functional relationship between SIRT3 genetic variation and myocardial infarction susceptibility, but not proving causality [281]. A more recent case-control study reported associations between SIRT3 rs11246029 T/C polymorphism, oxidative-stress markers, and CAD susceptibility [255]. The small sample size and observational design support an associative interpretation only. These studies do not establish SIRT3 dysfunction as a primary cause of CAD, but they justify discussing SIRT3 as a candidate genetic and mitochondrial redox-modulating factor in ischemic coronary disease. SIRT6 has also been examined in human CAD. In a Chinese Han population, SIRT6 tagSNPs rs352493 and rs3760908 were associated with angiographic CAD severity independently of conventional risk factors [254]. This finding supports a population-specific association with disease severity, not proof that SIRT6 variation directly causes CAD.

#### 5.9.2. Preclinical and Mechanistic Evidence

In endothelial cells, SIRT1 deacetylates endothelial nitric oxide synthase (eNOS), increases eNOS activity, and promotes nitric oxide-mediated endothelium-dependent vasodilation [282]. This mechanism is relevant to CAD because endothelial dysfunction and impaired nitric oxide bioavailability are early features of atherogenesis, but the evidence derives primarily from endothelial and vascular models.

SIRT1 effects in CAD-relevant cells are context-dependent. In EPCs, miR-34a suppresses SIRT1 expression and contributes to an inverse miR-34a/SIRT1 pattern observed in CAD patients [279]. These data provide a mechanistic explanation for the inverse miR-34a/SIRT1 pattern observed in CAD patients, but the experiments do not show that this pathway alone determines CAD progression in humans [279]. In VSMCs, C2dat1 acts as a competing endogenous RNA for miR-34a-5p, increases SIRT1 expression, and promotes VSMC proliferation and migration after stimulation with platelet-derived growth factor-BB or tumor necrosis factor-α [280]. Therefore, reduced SIRT1 in monocytes or endothelial-lineage cells may accompany inflammatory or endothelial dysfunction, whereas ncRNA-driven SIRT1 upregulation in VSMCs may support proliferative remodeling in specific vascular niches.

SIRT3 provides a plausible mechanistic link to CAD because it regulates key mitochondrial processes, including oxidative phosphorylation, fatty-acid oxidation, and antioxidant defense. SIRT3 deacetylates long-chain acyl-CoA dehydrogenase and supports mitochondrial fatty-acid oxidation, and it deacetylates superoxide dismutase 2 (SOD2) to enhance mitochondrial superoxide detoxification [118,283]. These mechanisms may modify redox and metabolic stress in ischemic coronary disease, but direct evidence that SIRT3 dysfunction initiates human CAD is lacking. SIRT6 may influence atherogenesis through hepatic lipid regulation and VSMC senescence. In hepatic metabolic models, FOXO3 and SIRT6 repress Pcsk9 transcription and reduce circulating low-density lipoprotein cholesterol [262]. In human and mouse plaque-derived VSMCs, reduced SIRT6 is associated with telomere dysfunction and senescence, whereas VSMC-specific SIRT6 overexpression in ApoE-deficient mice reduces atherosclerosis and plaque-cell senescence through a deacetylase-dependent mechanism [284]. These findings support a protective role for SIRT6 in experimental atherosclerosis but should not be extrapolated directly to human coronary outcomes. Both stable and unstable forms of CAD often culminate in myocardial infarction, where reperfusion therapies can trigger IRI. In this setting, myocardial sirtuin activity is a key determinant of infarct size and post-ischemic recovery. In experimental cardiac IRI, cardiomyocyte SIRT1 is protective. Cardiac-specific Sirt1 deletion increases susceptibility to IRI, leading to larger infarcts and worse left ventricular function. Conversely, moderate cardiac overexpression of SIRT1 reduces infarct size, in part by activating FOXO1-dependent antioxidant pathways (e.g., MnSOD induction) and suppressing Bax/caspase-3-mediated apoptosis [190]. SIRT6 also protects cardiomyocytes in IRI models by activating FOXO3α-dependent antioxidant signaling through an AMPK-dependent pathway [103].

Mitochondrial sirtuins further modulate cardiomyocyte survival during IRI. SIRT3 deficiency impairs post-ischemic recovery and increases myocardial ROS generation in experimental models. Interventions that enhance NAD^+^–SIRT3 signaling attenuate IRI by limiting mitochondrial oxidative stress and preserving mitochondrial function [285]. SIRT4 overexpression attenuates myocardial IRI by reducing apoptosis and preserving mitochondrial function, although separate pressure-overload studies indicate that SIRT4 can promote pathological remodeling, supporting a context-dependent role [203,246]. SIRT5 deficiency increases infarct size in ex vivo cardiac IRI, consistent with a protective role for mitochondrial desuccinylation and succinylome control in the ischemic heart [205]. Overall, preclinical data support cardioprotective roles for SIRT1, SIRT3, SIRT5, and SIRT6 in IRI models, whereas clinical evidence that therapeutic modulation of sirtuins improves MI or CAD outcomes remains limited.

### 5.10. Role of Sirtuins in Hypertension

Hypertension is a major, modifiable cardiovascular risk factor that affects more than one billion adults worldwide. It is traditionally defined by persistently elevated systolic blood pressure ≥ 140 mmHg and/or diastolic blood pressure ≥ 90 mmHg, although some recent guidelines use lower thresholds [286]. Hypertensive vascular injury involves the interplay of endothelial dysfunction, VSMC remodeling, oxidative stress, and neurohumoral activation. Sirtuins may modulate several of these processes through NAD^+^-dependent deacylation of histone and non-histone proteins, but most evidence in hypertension remains preclinical [287].

#### 5.10.1. Clinical and Human Evidence

Human evidence directly linking sirtuins to systemic hypertension is limited. In patients with essential hypertension, urinary SIRT1 levels were lower in those with albuminuria than in normoalbuminuric hypertensive patients and were associated with urinary albumin excretion, supporting urinary SIRT1 as a candidate noninvasive marker of early hypertensive renal injury rather than a proven causal mediator [288]. In human vascular samples and hypertension-related studies, reduced SIRT3 expression or activity has been associated with vascular oxidative stress and inflammation, but available clinical data do not establish that SIRT3 deficiency initiates essential hypertension [97,267]. No adequately powered clinical trial has shown that pharmacological activation of SIRT1, SIRT3, or SIRT6 lowers blood pressure or prevents hypertensive target-organ damage in patients.

#### 5.10.2. Preclinical and Mechanistic Evidence

SIRT1 is the best-characterized sirtuin in experimental systemic hypertension. SIRT1 expressed in VSMCs and endothelial cells is essential for maintaining normal vascular tone and structure. In mice, VSMC-specific SIRT1 overexpression attenuates angiotensin II-induced increases in systolic blood pressure, vascular hypertrophy, collagen deposition, and fibrosis, as well as improves aortic compliance, indicating that VSMC SIRT1 restrains angiotensin II-driven vascular remodeling in this model [289]. SIRT1 also modulates angiotensin II signaling in VSMCs by downregulating angiotensin II type 1 receptor expression, providing an additional mechanism by which SIRT1 may reduce angiotensin II responsiveness [290]. In Klotho-deficient mice, reduced vascular SIRT1 activity is associated with arterial stiffness and hypertension. SIRT1 activation with SRT1720 improves aortic compliance, reduces pulse wave velocity, enhances AMPK–endothelial nitric oxide synthase (eNOS) signaling, and lowers blood pressure [291]. In angiotensin II-infused mice, nicotinamide phosphoribosyltransferase (NAMPT) preserves vascular NAD^+^ availability and SIRT1 activity. NAMPT deficiency increases vascular reactive oxygen species (ROS), mitogen-activated protein kinase activation, vascular remodeling, and hypertension, whereas NAMPT restoration or NAD^+^ supplementation attenuates these responses [292]. These data support a protective vascular role for the NAMPT–NAD^+^–SIRT1 axis in angiotensin II-dependent hypertension, but they do not prove therapeutic efficacy in human essential hypertension.

SIRT3 regulates hypertension-related vascular injury primarily through mitochondrial redox control. In experimental hypertension, Sirt3 deficiency or depletion increases mitochondrial superoxide, reduces endothelial nitric oxide bioavailability, worsens endothelial dysfunction, and augments the hypertensive response to angiotensin II and deoxycorticosterone acetate-salt [97,267]. Conversely, Sirt3 overexpression in mice decreases vascular superoxide production, improves endothelium-dependent relaxation, and attenuates vascular remodeling and end-organ injury. Mechanistically, angiotensin II-induced hypertension is associated with SIRT3 impairment, SOD2 hyperacetylation, reduced SOD2 activity, and increased mitochondrial ROS; mitochondrial catalase prevents SIRT3/SOD2 impairment and attenuates hypertension in this model [97]. Beyond large arteries, SIRT3 also protects the renal microvasculature. In angiotensin II-induced hypertension, SIRT3 downregulation in renal endothelial cells is accompanied by endothelial-to-mesenchymal transition, increased ROS, and renal fibrosis. Restoration of SIRT3 activates a FOXO3a–catalase pathway that suppresses endothelial-to-mesenchymal transition, limits interstitial fibrosis, and preserves kidney function despite elevated blood pressure [97,293]. These findings situate SIRT3 as a mitochondrial antioxidant regulator of vascular and renal injury in experimental hypertension.

SIRT has recently been implicated in endothelial control of blood pressure and hypertensive cardiorenal injury. Endothelial-specific deletion of Sirt6 in mice resulted in elevated systolic blood pressure, impaired endothelium-dependent relaxation, and more severe cardiac and renal injury during angiotensin II- or DOCA-salt-induced hypertension [294]. Mechanistically, endothelial SIRT6 helps maintain vasodilator function and vascular NO bioavailability through an Nkx3.2–GATA5 transcriptional pathway. SIRT6 deacetylates histone H3K9 at the Nkx3.2 locus, repressing Nkx3.2 transcription; this relieves inhibition of GATA5 expression, a regulator of blood pressure and endothelial function, thereby supporting GATA5-mediated endothelial protection [294,295]. Loss of SIRT6 disrupts this Nkx3.2–GATA5 pathway, promoting endothelial dysfunction, vascular inflammation, and fibrosis, and exacerbating cardiorenal injury in hypertensive mice. SIRT6 overexpression or activation reverses these changes in vivo [294]. These vascular effects are consistent with broader data showing that SIRT6 suppresses NF-κB-dependent vascular inflammation, oxidative stress, and fibrotic signaling in multiple tissues, and support the biological plausibility of these mechanisms, but these mechanisms should be presented as experimental rather than clinically validated antihypertensive pathways [63,296].

Overall, preclinical studies identify SIRT1, SIRT3, and SIRT6 as regulators of vascular tone, arterial stiffness, oxidative stress, endothelial function, and hypertensive target-organ injury. SIRT1 mainly modulates angiotensin II responsiveness, NAD^+^-dependent redox signaling, and AMPK–eNOS signaling; SIRT3 limits mitochondrial ROS through SOD2- and FOXO3a-related antioxidant pathways; and SIRT6 preserves endothelial nitric oxide signaling through chromatin-dependent regulation of the Nkx3.2–GATA5 axis. Current human evidence is insufficient to conclude that reduced sirtuin activity causes essential hypertension or that sirtuin activation improves clinical outcomes. The summary of major reported cardiovascular effects is presented in Table 2.

## 6. Therapeutic Modulation of Sirtuins in Cardiovascular Disease

The pivotal roles of sirtuins in redox homeostasis, mitochondrial metabolism, inflammatory signaling, and fibrosis have made these NAD^+^-dependent deacylases attractive drug targets in CVD. Pharmacological strategies to modulate sirtuin activity fall broadly into three categories: small-molecule activators (natural products and synthetic sirtuin-activating compounds), interventions that raise intracellular NAD^+^ and thereby enhance sirtuin flux, and isoform-selective inhibitors, used in settings where chronic or excessive sirtuin activity is maladaptive [12]. Because most CVD phenotypes arise from chronic, multi-hit injury, the therapeutic window, isoform selectivity, and tissue distribution of each modulator are critical determinants of efficacy and safety.

### 6.1. Natural Sirtuin Activators with Cardiovascular Actions

Polyphenolic SIRT1 activators are the best-characterized natural sirtuin modulators. Resveratrol, a stilbene found in grapes and red wine, was initially described as an allosteric SIRT1 activator that extends lifespan in lower organisms. In cardiovascular models, resveratrol increases SIRT1-dependent deacetylation of eNOS and PGC-1α, improves endothelial NO bioavailability, and attenuates cardiac dysfunction in pressure overload, ischemia–reperfusion, and DCM [192]. Small randomized trials in patients with type 2 diabetes or CAD indicate that resveratrol can improve metabolic parameters, increase SIRT1 expression in peripheral blood mononuclear cells, and enhance antioxidant capacity, although effects on blood pressure, vascular function, and hard cardiovascular endpoints remain inconsistent [304]. Low oral bioavailability, rapid metabolism, and pleiotropic target engagement limit straightforward translation. Other plant polyphenols structurally related to resveratrol, such as quercetin, fisetin, and butein, also upregulate SIRT1 in vitro and in vivo. Quercetin improves endothelial function, attenuates oxidative stress, and modulates lipid metabolism in experimental models, supporting its anti-atherosclerotic and antihypertensive potential, while curcumin increases SIRT1 and SIRT3 expression, enhances mitochondrial function, and mitigates cardiac remodeling through activation of AMPK and Nrf2 signaling pathways [174,305].

Beyond SIRT1-targeted molecules, several natural compounds preferentially engage mitochondrial sirtuins. The resveratrol glucoside polydatin enhances SIRT3 activity, preserves mitochondrial integrity, and improves cardiac function in rodent models of DCM and radiation-induced cardiac injury [306]. Honokiol, a lignan from Magnolia bark, is a direct SIRT3 agonist that improves mitochondrial respiration, reduces ROS production, and protects against cardiac hypertrophy and IRI [307]. These data highlight mitochondrial sirtuins, particularly SIRT3, as tractable targets for cardioprotection.

In addition to polyphenols and lignans, other redox-active small molecules may influence sirtuin-related pathways. Pyrroloquinoline quinone (PQQ), a naturally occurring quinone present in various foods, has been shown to regulate mitochondrial function and cellular redox homeostasis [308,309]. Experimental studies demonstrate that PQQ promotes mitochondrial biogenesis and improves mitochondrial efficiency, at least in part through pathways involving PGC-1α [308]. In parallel, PQQ exhibits antioxidant properties and reduces reactive oxygen species (ROS) levels, thereby supporting mitochondrial integrity and cellular metabolic function [309]. Although PQQ has not been identified as a direct activator of sirtuins, its effects on mitochondrial function and oxidative stress may indirectly influence sirtuin-regulated pathways, particularly those involving mitochondrial sirtuins such as SIRT3 [309]. However, the extent and specificity of this interaction remain insufficiently defined. Overall, while PQQ shows promise as a modulator of mitochondrial homeostasis, current evidence is largely limited to experimental models, and its role in cardiovascular disease and sirtuin-targeted therapy requires further investigation.

Despite promising preclinical efficacy, most natural activators share several limitations: modest potency at achievable plasma concentrations, extensive off-target actions, and suboptimal pharmacokinetics. These features complicate dose-response relationships and motivate the search for more potent, isoform-selective small molecules.

### 6.2. Synthetic Sirtuin-Activating Compounds and Isoform-Selective Agonists

High-throughput screening and medicinal chemistry have yielded several classes of synthetic sirtuin-activating compounds (STACs). Early agents such as SRT1720, SRT1460, and SRT2104 were developed as allosteric SIRT1 activators and improve insulin sensitivity, mitochondrial biogenesis, and lipid metabolism in diet-induced and genetic obesity models via SIRT1–PGC-1α signaling [310]. In humans, short-term SRT2104 administration in healthy volunteers, elderly individuals, and patients with type 2 diabetes has shown acceptable safety and modest improvements in lipid profile and some vascular or inflammatory biomarkers, but no consistent benefit on glycemic control or major cardiovascular outcomes, and pharmacokinetics are variable [311,312]. Moreover, subsequent work has highlighted that the apparent SIRT1 activation by STACs is substrate- and assay-dependent, so they are best viewed as SIRT1-biased modulators rather than universal activators.

More recently, attention has shifted toward isoform-selective activators beyond SIRT1. The pyrrolo[1,2-a]quinoxaline derivative UBCS039 and the bis-benzenesulfonamide prodrug MDL-800 enhance SIRT6 deacetylase activity and reduce H3K9ac and H3K56ac at SIRT6 target loci. In fibroblasts and hepatic stellate cells, MDL-800 suppresses TGF-β–Smad signaling and ECM gene expression, effectively recapitulating the antifibrotic and antihypertrophic properties attributed to SIRT6 in the heart and vasculature [1,313]. Direct in vivo cardiac data for these SIRT6 activators remain limited, but their ability to remodel chromatin at proinflammatory and profibrotic loci makes them compelling candidates for further testing in models of cardiac and vascular remodeling. In parallel, SIRT3-directed molecules inspired by honokiol, along with new chemotypes designed to exploit acyl-lysine specificities (e.g., desuccinylation and demalonylation for SIRT5), are being developed to selectively target mitochondrial sirtuins [314,315].

Given the context-dependent roles of SIRT3, SIRT4, and SIRT5 in oxidative stress signaling and cell death, careful titration of activity and attention to tissue specificity will be crucial to avoid tipping adaptive stress responses into maladaptive damage.

### 6.3. NAD^+^-Boosting Strategies as Indirect Pan-Sirtuin Activators

Because all sirtuins depend on NAD^+^ as a cosubstrate, augmenting NAD^+^ availability is an attractive complementary approach [316]. NAD^+^ levels can be increased by supplying precursors in the salvage pathway, such as nicotinamide riboside (NR) or nicotinamide mononucleotide (NMN), or by inhibiting NAD-consuming enzymes such as CD38. In murine models of HF and pressure overload, NAD^+^ replenishment via NMN or related precursors restores myocardial NAD^+^, preserves mitochondrial homeostasis, improves contractile function, and reduces ventricular remodeling. These benefits are markedly attenuated in SIRT3-deficient hearts, indicating that part of the cardioprotective effect is sirtuin-dependent [317,318].

Clinical studies have begun to translate these findings. Oral NMN supplementation increases blood NAD^+^ in healthy adults and older men with an acceptable safety profile [319,320,321]. In patients with HF with preserved ejection fraction (HFpEF), short-term nicotinamide administration improved exercise capacity and indices of diastolic function, consistent with a role for myocardial NAD^+^ depletion in HFpEF pathophysiology [322,323]. Nicotinamide riboside has also been tested in small pilot trials in systolic and diastolic HF and in older adults with cardiometabolic risk, with evidence of NAD^+^ repletion and modest improvements in some vascular endpoints, though effects on blood pressure and clinical outcomes remain preliminary [324]. From a cardiovascular standpoint, NAD^+^ boosters have the advantage of simultaneously engaging nuclear and mitochondrial sirtuins, thereby potentially improving endothelial function, attenuating oxidative and inflammatory injury, and stabilizing mitochondrial energetics. However, they lack isoform selectivity and also increase the activity of non-sirtuin NAD-dependent enzymes (PARPs, CD38). Therefore, long-term cardiovascular safety and durability of benefit still need to be established.

### 6.4. Sirtuin Inhibition and the “Double-Edged Sword” Problem

Although most cardiovascular applications aim to enhance sirtuin activity, selective inhibition may be desirable when chronic overactivation contributes to pathology. The SIRT1 inhibitor EX-527 (selisistat) and dual SIRT1 and SIRT2 inhibitors such as cambinol, sirtinol, splitomicin, and tenovin-6 were developed primarily for oncology but are widely used as mechanistic probes [310]. Cardiac studies using EX-527 demonstrate that both systemic SIRT1 deficiency and excessive SIRT1 activation can aggravate pressure overload-induced hypertrophy and HF, underscoring the narrow therapeutic window of SIRT1 in the heart and the risk of indiscriminate, high-level activation [213]. For SIRT2, chemotypes such as SirReal2 and AGK2 are potent and selective inhibitors being evaluated mainly in cancer and neurodegeneration. Given that SIRT2 influences NFAT and AMPK signaling in cardiomyocytes and vascular cells, these molecules could, in principle, be repurposed to modulate pathological hypertrophy or vascular remodeling, but cardiovascular efficacy and safety data are essentially lacking [325]. SIRT5 inhibition by suramin and related compounds is well characterized biochemically. However, strong experimental evidence that SIRT5 protects against cardiac IRI and adverse remodeling through desuccinylation of key mitochondrial enzymes argues against broad SIRT5 blockade in CVD [326]. These examples highlight a broader theme—sirtuins often exert biphasic or context-dependent effects, especially in the heart. Therapeutic strategies must therefore consider disease stage, cell type, and systemic inflammatory status rather than assuming that maximal activation or inhibition will be uniformly beneficial.

Overall, current data support several complementary strategies for sirtuin-based interventions in CVD. First, NAD^+^ availability can be augmented to boost endogenous sirtuin activity, particularly in conditions such as HFpEF and cardiometabolic HF, where myocardial NAD^+^ depletion is evident [317,322,324]. Second, isoform-selective activators or stabilizers can be developed, for example, compounds that enhance SIRT3- and SIRT5-dependent mitochondrial quality control or SIRT6-mediated antifibrotic and anti-inflammatory chromatin remodeling. And finally, sirtuin-centered regulatory axes can be targeted with RNA-based approaches, including modulation of microRNAs and lncRNAs that control SIRT1 and SIRT6 expression and thereby autophagy, oxidative stress, and fibrosis.

## 7. Translational Considerations and Future Directions

Current evidence points to SIRT1, SIRT3, and SIRT6 as the most promising near-term targets for cardiovascular therapy. Upregulation of these isoforms, through small-molecule activators or NAD^+^ restoration, consistently improves mitochondrial function, reduces oxidative and inflammatory stress, and limits adverse remodeling in preclinical models of atherosclerosis, HF, and hypertension. Natural polyphenols such as resveratrol and related compounds provided the first proof of principle that pharmacological sirtuin activation can improve endothelial function and cardiometabolic profiles, but their modest potency, poor bioavailability, and pleiotropic actions limit their stand-alone utility. Synthetic SIRT1-biased STACs and SIRT6-selective activators offer better drug-like properties and show favorable metabolic and antifibrotic effects in early studies, yet robust cardiovascular efficacy and long-term safety still need to be established. NAD^+^-boosting strategies (nicotinamide, NR, NMN, and CD38 inhibition) are already in early-phase clinical evaluation in HF and cardiometabolic risk states. They appear safe over the short term and effectively raise NAD^+^, but optimal dosing, chronic safety, interactions with guideline-directed therapies, and impact on clinical cardiovascular endpoints remain open questions. In parallel, the development of truly isoform-selective modulators of mitochondrial sirtuins and of inhibitors for settings in which sirtuin activity is maladaptive (e.g., excessive SIRT1 in some hypertrophic states) is still at an early stage, particularly for SIRT4, SIRT5, and SIRT7. Translational progress is also limited by heterogeneous experimental models, off-target effects, and a current reliance on small human studies focused on surrogate biomarkers rather than hard outcomes. Going forward, combination approaches (e.g., pairing sirtuin modulators with renin–angiotensin–aldosterone system blockers, lipid-lowering agents, or modern antidiabetic drugs) and precision strategies that integrate pharmacology, human genetics, and high-resolution cardiovascular phenotyping will likely be required to identify the right target, dose, and patient population.

## 8. Conclusions

Sirtuins constitute a highly conserved family of NAD^+^-dependent enzymes that connect cellular energy status with chromatin regulation, mitochondrial function, redox homeostasis, inflammation, autophagy, apoptosis, and metabolic adaptation. Their biological effects are strongly determined by isoform-specific enzymatic activity, subcellular localization, tissue context, disease stage, and the nature of the cellular stressor. In cardiovascular biology, the most consistent evidence supports protective roles for SIRT1, SIRT3, and SIRT6 in maintaining endothelial function, mitochondrial integrity, antioxidant defense, genomic stability, and resistance to adverse remodeling. However, these effects are not universal, and excessive or context-inappropriate activation may be maladaptive.

Current evidence indicates that sirtuin pathways are involved in several cardiovascular conditions, including metabolic and diabetic cardiomyopathy, sepsis-induced myocardial dysfunction, alcoholic cardiomyopathy, myocardial ischemia-reperfusion injury, cardiac hypertrophy, fibrosis, heart failure, atherosclerosis, coronary artery disease, and hypertension. Across these settings, sirtuins act less as isolated disease-specific mediators and more as regulatory nodes integrating metabolic stress, mitochondrial dysfunction, oxidative injury, inflammatory signaling, and tissue repair. Importantly, the roles of SIRT2, SIRT4, SIRT5, and SIRT7 remain more context-dependent and less clinically defined, requiring cautious interpretation.

Although pharmacological activation of sirtuins, NAD^+^ restoration, and isoform-selective modulation are promising therapeutic strategies, clinical translation remains at an early stage. Most available evidence is derived from experimental models, while human data are largely associative or based on small studies using surrogate endpoints. Future research should prioritize isoform-selective interventions, robust cardiovascular phenotyping, long-term safety assessment, and well-designed clinical trials to determine whether modulation of sirtuin pathways can improve cardiovascular outcomes. Overall, sirtuins represent biologically plausible and mechanistically rich therapeutic targets, but their clinical use will require precision approaches that account for isoform specificity, disease context, timing, and patient phenotype.

## Figures and Tables

**Table 1:** Subcellular localization, enzymatic activities and principal cellular functions of mammalian sirtuins.

Sirtuin	Predominant Localization	Main Enzymatic Activity	Principal Mechanisms/Functions
SIRT1	Nucleus; shuttles to cytoplasm	NAD^+^-dependent deacetylase	p53 deacetylation; FOXO regulation; PGC-1α activation; NF-κB/RelA repression; regulation of DNA repair, oxidative stress, inflammation, apoptosis, and metabolism
SIRT2	Cytoplasm; nuclear during mitosis	NAD^+^-dependent deacetylase	α-tubulin deacetylation; H4K16 deacetylation during mitosis; regulation of cytoskeletal dynamics, cell-cycle progression, genome integrity, and metabolic adaptation
SIRT3	Mitochondria	Major mitochondrial NAD^+^-dependent deacetylase	Deacetylation of mitochondrial metabolic enzymes; activation of fatty-acid oxidation; regulation of tricarboxylic acid cycle activity, oxidative phosphorylation, ATP production, and SOD2/MnSOD-dependent antioxidant defense
SIRT4	Mitochondrial matrix	ADP-ribosyltransferase; context-dependent deacylase	Inhibition of glutamate dehydrogenase; regulation of amino-acid-stimulated insulin secretion, glutamine metabolism, fatty-acid oxidation, leucine metabolism, ATP homeostasis, redox balance, and mitochondrial stress responses
SIRT5	Mainly mitochondria; also cytosolic in some contexts	Desuccinylase; demalonylase; deglutarylase	Removal of negatively charged lysine acyl modifications; regulation of mitochondrial protein succinylation, fatty-acid oxidation, tricarboxylic acid cycle enzymes, oxidative phosphorylation, urea cycle activity, and NADPH-dependent antioxidant defense
SIRT6	Nucleus; chromatin-associated	Deacetylase; mono-ADP-ribosyltransferase; long-chain fatty-acyl deacylase	H3K9 and H3K56 deacetylation; PARP1 activation; NF-κB target-gene repression; regulation of DNA repair, telomere maintenance, chromatin accessibility, glucose and lipid metabolism, inflammation, and stress responses
SIRT7	Nucleolus; nucleoplasm/chromatin in selected contexts	NAD^+^-dependent deacetylase; RNA-activated deacylase; histone desuccinylase	Regulation of RNA polymerase I-dependent rRNA transcription; ribosome biogenesis; H3K122 desuccinylation; chromatin compaction; DNA double-strand break repair; context-dependent regulation of stress responses and remodeling

Abbreviations: ADP, adenosine diphosphate; ATP, adenosine triphosphate; H3K9, histone H3 lysine 9; H3K56, histone H3 lysine 56; H3K122, histone H3 lysine 122; H4K16, histone H4 lysine 16; MnSOD, manganese superoxide dismutase; NAD^+^, nicotinamide adenine dinucleotide, oxidized form; NADPH, nicotinamide adenine dinucleotide phosphate, reduced form; PARP1, poly(ADP-ribose) polymerase 1; PGC-1α, peroxisome proliferator-activated receptor gamma coactivator 1-alpha; RNA, ribonucleic acid; SIRT, sirtuin; SOD2, superoxide dismutase 2.

**Table 2:** Main cardiovascular effects of mammalian sirtuins.

Sirtuin	Main Cardiovascular Effects	Evidence Strength/Caution	Ref.
SIRT1	Supports endothelial nitric oxide signaling; reduces endothelial activation, oxidative stress, inflammation, and apoptosis; attenuates atherosclerosis and experimental ischemia–reperfusion injury when activity is physiological or moderately enhanced.	Strongest cardiovascular evidence among sirtuins, mainly preclinical with supportive human association data; excessive cardiac SIRT1 activity may aggravate remodeling, so effects are dose- and context-dependent.	[13,297]
SIRT2	Regulates cardiac stress responses, hypertrophic signaling, oxidative stress, and vascular inflammation; reported effects differ between models, with both protective and maladaptive roles described.	Evidence is mainly experimental and inconsistent; SIRT2 should not be classified as uniformly cardioprotective or harmful.	[245,298]
SIRT3	Preserves mitochondrial function, fatty-acid oxidation, ATP production, and antioxidant defense; attenuates cardiac hypertrophy, heart failure remodeling, vascular oxidative stress, and myocardial ischemia–reperfusion injury in experimental models.	Best-supported mitochondrial sirtuin in cardiovascular disease; evidence is strong preclinically, but clinical therapeutic validation remains limited.	[299,300,301]
SIRT4	Limits apoptosis and mitochondrial injury in acute myocardial ischemia–reperfusion models, but promotes hypertrophy, fibrosis, and heart failure progression during chronic Ang II or pressure-overload stress.	Strongly context-dependent; acute injury and chronic remodeling data point in opposite directions.	[203,204]
SIRT5	Regulates mitochondrial desuccinylation and energy metabolism; reduces susceptibility to myocardial ischemia–reperfusion injury and may support adaptation to pressure-overload stress.	Evidence is mainly preclinical; protective effects are linked to succinylome control and mitochondrial metabolism, but model differences remain important.	[205]
SIRT6	Protects endothelial function, suppresses vascular inflammation and senescence, limits experimental atherosclerosis, attenuates cardiac hypertrophy and fibrosis, and reduces hypertensive cardiorenal injury in mouse models.	Strong preclinical evidence; human data are mostly associative, including reduced SIRT6 in some cardiovascular disease settings.	[294,302,303]
SIRT7	Maintains long-term myocardial homeostasis; SIRT7 deficiency causes cardiac hypertrophy, apoptosis, mitochondrial dysfunction, and inflammatory cardiomyopathy in mice; effects in fibrosis and acute injury are cell- and timing-dependent.	Evidence is mainly experimental; SIRT7 should be described as context-dependent, especially in fibroblast activation, infarct repair, and acute ischemic injury.	[175]

Abbreviations: Ang II, angiotensin II; SIRT, sirtuin.

## Data Availability

No new data were created or analyzed in this study.

## Abbreviations

The following abbreviations are used in this manuscript:

ACE2	angiotensin-converting enzyme 2
ADP	adenosine diphosphate
Akt	protein kinase B
AMPK	AMP-activated protein kinase
ApoE	apolipoprotein E
ATP	adenosine triphosphate
ATG5, ATG7, ATG8	autophagy-related 5, 7, 8
BAX	BCL2-associated X protein
Bcl-2	B-cell lymphoma 2
Bcl-xL	B-cell lymphoma-extra large
Bnip3	BCL2/adenovirus E1B 19 kDa-interacting protein 3
BRCA1	breast cancer 1
CAD	coronary artery disease
CCR7	C-C chemokine receptor 7
CD38	cluster of differentiation 38
CHMP2B	charged multivesicular body protein 2B
CREB	cAMP response element-binding protein
CTRP1	C1q/tumor necrosis factor-related protein 1
CVD	cardiovascular disease
DCM	diabetic cardiomyopathy
eNOS	endothelial nitric oxide synthase
ERK1/2	extracellular signal-regulated kinase 1/2
ESR	estrogen receptor
EZH2	enhancer of zeste homolog 2
FGF21	fibroblast growth factor 21
Fas	Fas cell-surface death receptor
FasL	Fas ligand
FOXO1, FOXO3, FOXO3a	forkhead box O1, O3, O3a
G6PD	glucose-6-phosphate dehydrogenase
GABPβ1	GA-binding protein beta 1
GLUT1, GLUT2, GLUT4	glucose transporter 1, 2, 4
HF	heart failure
HFpEF	heart failure with preserved ejection fraction
HIF	hypoxia-inducible factor
HIPK2	homeodomain-interacting protein kinase 2
HO-1	heme oxygenase 1
IDH2	isocitrate dehydrogenase 2
IGF	insulin-like growth factor
IL-	interleukin-
IRI	ischemia–reperfusion injury
JNK	c-Jun N-terminal kinase
Keap1	Kelch-like ECH-associated protein 1
LARP7	La-related protein 7
LC3	microtubule-associated protein 1 light chain 3
LCAD	long-chain acyl-CoA dehydrogenase
LDL	low-density lipoprotein
LKB1	liver kinase B1
LOX-1	lectin-like oxidized LDL receptor 1
LXR	liver X receptor
MAPK	mitogen-activated protein kinase
MnSOD	manganese superoxide dismutase
mPTP	mitochondrial permeability transition pore
mRNA	messenger RNA
mTOR	mechanistic target of rapamycin
NAD^+^	nicotinamide adenine dinucleotide
NADH	reduced nicotinamide adenine dinucleotide
NADPH	nicotinamide adenine dinucleotide phosphate
NAMPT	nicotinamide phosphoribosyltransferase
NBS1	Nijmegen breakage syndrome 1
NFAT	nuclear factor of activated T cells
NF-κB	nuclear factor kappa B
NLRP3	NOD-like receptor family pyrin domain-containing 3
NMN	nicotinamide mononucleotide
NO	nitric oxide
Nrf2	nuclear factor erythroid 2-related factor 2
OPA1	optic atrophy 1
oxLDL	oxidized low-density lipoprotein
PAI-1	plasminogen activator inhibitor 1
PARP-1	poly(ADP-ribose) polymerase 1
PCSK9	proprotein convertase subtilisin/kexin type 9
PGC-1α	peroxisome proliferator-activated receptor gamma coactivator 1-alpha
PI3K	phosphoinositide 3-kinase
PINK1	PTEN-induced kinase 1
PKM2	pyruvate kinase M2
PPARα	peroxisome proliferator-activated receptor alpha
ROS	reactive oxygen species
SHMT2	serine hydroxymethyltransferase 2
SIR2	silent information regulator 2
SIRT1–SIRT7	sirtuin 1–sirtuin 7
SMAD4	SMAD family member 4
SOD2	superoxide dismutase 2
SREBP	sterol regulatory element-binding protein
SREBPs	sterol regulatory element-binding proteins
STAT3	signal transducer and activator of transcription 3
STACs	sirtuin-activating compounds
TCA	tricarboxylic acid
TFEB	transcription factor EB
TGF-β	transforming growth factor beta
TOM1	target of myb1 membrane trafficking protein 1
TNF-α	tumor necrosis factor alpha
VSMC	vascular smooth muscle cell
Wnt	Wingless/Integrated
